# Multigraded Heterojunction Hole Extraction Layer of ZIF‐Co_*x*_Zn_1−*x*_ on Co_3_O_4_/TiO_2_ Skeleton for a New Photoanode Architecture in Water Oxidation

**DOI:** 10.1002/smsc.202000033

**Published:** 2021-02-24

**Authors:** Rui Tang, Lizhuo Wang, Meihui Ying, Wenjie Yang, Amanj Kheradmand, Yijiao Jiang, Zhiyun Li, Yi Cui, Rongkun Zheng, Jun Huang

**Affiliations:** ^1^ School of Physics Sydney Nano Institute The University of Sydney Sydney NSW 2006 Australia; ^2^ School of Chemical and Biomolecular Engineering Sydney Nano Institute The University of Sydney NSW 2037 Australia; ^3^ School of Engineering Macquarie University Sydney NSW 2109 Australia; ^4^ Vacuum Interconnected Nanotech Workstation Suzhou Institute of Nano–Tech and Nano-Bionics The Chinese Academy of Sciences Suzhou 215123 China

**Keywords:** carrier separation, metal-organic frameworks, multigraded heterojunctions, photoelectrochemical water oxidation, ZIF-Co_*x*_Zn_1−*x*_/Co_3_O_4_/TiO_2_

## Abstract

Multigraded heterojunction (GHJ) attracts increasing attention in solar energy conversion fields due to the controllable carrier transport process. However, it is hard to coordinately manipulate the components and band structure of the photoelectrochemical (PEC) catalyst. Despite the adjustable composition of the metal‐organic framework (MOF), energy band‐engineered MOF materials are rarely used in PEC systems subject to their unclear energy band structure. Herein, a brand new photoanode architecture with MOF‐based multi‐GHJ as the hole extraction channel exhibits outstanding PEC water oxidation performance. ZIF‐Co_*x*_Zn_1−*x*_ multi‐GHJs with continuously adjustable energy band structures are obtained by tailoring Co/Zn ratio. ZIF‐Co_*x*_Zn_1−*x*_ multi‐GHJ‐modified Co_3_O_4_/TiO_2_ photoanodes (ZIF‐Co_*x*_Zn_1−*x*_/Co_3_O_4_/TiO_2_) exhibit a greatly facilitated carrier separation. ZIF‐Co further causes improved interfacial carrier injection as the water oxidation cocatalyst. By constructing the network‐like Co_3_O_4_ skeleton, mass transport and light capture processes are also enhanced. With the synergy of energy band engineering and nanostructure design, the 4‐grade GHJ/Co_3_O_4_/TiO_2_ photoanode shows an excellent photocurrent density (2.91 mA cm^−2^ at 1.23 V vs. reversible hydrogen electrode) and carrier migration efficiency (73.3%), which are 312% and 554% higher than intrinsic TiO_2_, respectively. Herein, new insights into energy band‐engineered MOF materials with excellent carrier separation/extraction, which is promising for PEC and other optoelectronic applications, are provided.

## Graphical abstract

Metal‐organic framework‐based multigraded heterojunction (GHJ) ZIF‐Co_*x*_Zn_1−*x*_/Co_3_O_4_/TiO_2_ photoanode for efficient photoelectrochemical water oxidation is developed. The introduction of ZIF‐Co_*x*_Zn_1−*x*_ the multi‐GHJ not only leads to an enhanced carrier separation efficiency but also causes enhanced surface reaction kinetics. Due to the unique network‐like Co_3_O_4_ skeleton, greatly improved mass transport and the light capture property are also harvested.

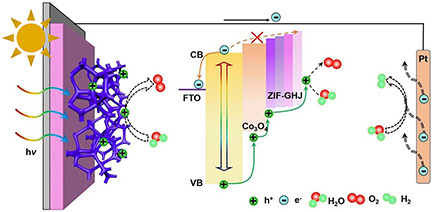

## 1 Introduction

Due to the sustainable energy‐environment challenge, the development of new clean energy from renewable sources has become increasingly important.^[^ ^1^, ^2^, ^3^ ^]^ Solar energy is the most naturally abundant, clean, and sustainable resource. Fujishima and Honda first reported that TiO_2_ can achieve effective photoelectrochemical (PEC) water splitting,^[^ ^4^ ^]^ which provides an alternative way to produce sustainable hydrogen. However, the overall water‐splitting reaction is challenged by the water oxidation half reaction due to its sluggish four‐proton multielectron process.^[^ ^5^, ^6^ ^]^ Generally, the photoinduced carriers have to go through a series of bulk/interfacial carrier recombination processes; then, the residue effective holes can participate in the water oxidation reaction.^[^ ^7^ ^]^ Only when the carrier recombination process can be efficiently suppressed, a better PEC water‐splitting performance can be achieved. At present, it is still a significant challenge to prepare photoanode materials with good carrier transport and interface hole injection efficiency.

To improve the carrier transport performance of semiconductors, the most commonly used strategy is the energy band engineering method, which is often used to suppress the recombination of photogenerated carriers.^[^ ^8^, ^9^ ^]^ However, previous works reported that the accumulation and recombination of electron–hole pairs occurred in the nondepleted layer region due to its relatively shallow depth.^[^ ^10^, ^11^ ^]^ Recently, Yin and coworkers demonstrated a promising method by constructing graded heterojunctions (GHJ) by sequentially depositing CsPbBr_*x*_I_3−*x*_ (*x *= 0, 2.7, 2.3, 2) layer by layer in perovskite solar cells and effectively widened the depth of the depletion layer.^[^ ^12^ ^]^ The GHJ provides an additional drift driving force to facilitate carrier separation compared with the typical type‐II heterojunction. So far, GHJ architecture is still mainly used in photovoltaic solar cells.^[^ ^13^, ^14^ ^]^ However, for conventional photoelectrocatalysts, it is difficult to construct GHJ photoanodes with a continuously adjustable energy band structure and composition simultaneously, which poses a serious challenge to achieving a high‐performance PEC water oxidation process. Therefore, it is of great significance to discover suitable photoelectrocatalysts with a tunable energy band structure and construct the GHJ‐based photoanode with a finely modulated energy band alignment.

Metal‐organic frameworks (MOF), such as ZIF‐67, ZIF‐8, and MIL‐125 (Ti), have been widely used in energy storage, gas adsorption, and other fields, thanks to their unique structure characteristics, such as a high specific surface area, rich pore channels, and metal core diversifications.^[^ ^15^ ^]^ On the other hand, MOFs are often used as precursors to prepare the derived photoelectrocatalyst, such as metal oxides,^[^ ^16^ ^]^ carbides,^[^ ^17^, ^18^ ^]^ and nitrides,^[^ ^19^ ^]^ so as to investigate their PEC performance. However, the preparation process of MOF‐derived catalysts will bring irreversible damage to the pore structure, which, to some extent, hinders their further application. As the semiconductor properties of MOF materials are not well defined, the research on MOF‐based photoelectrocatalysts is rarely reported. Therefore, it is of significant interest to explore the semiconductor properties of MOFs, aiming toward high‐performance PEC water oxidation systems.

Herein, for the first time, we demonstrate a simple hydrothermal electrodeposition method to prepare a MOF‐based multi‐GHJ architecture ZIF‐Co_*x*_Zn_1−*x*_/Co_3_O_4_/TiO_2_ photoanode for high‐performance PEC water oxidation. Based on Mott–Schottky methods, the energy band structures of ZIF‐Co_*x*_Zn_1−*x*_ are investigated. Through photoluminescence (PL) spectroscopy and PEC characterizations, the effects of multi‐GHJs on photogenerated carrier separation and transfer processes are systematically studied. Meanwhile, the function of the unique composition of the MOF‐based multi‐GHJ on interfacial carrier injection performance is also discussed. By comparing the mass transport and light‐harvesting performance of the network‐like photoanode and conventional planar‐structured photoanode, a deeper discussion and understanding of the influence of the photoanode microstructure on the photoelectrocatalytic performance has been proposed.

## 2 Results and Discussion

**Figure 1:**
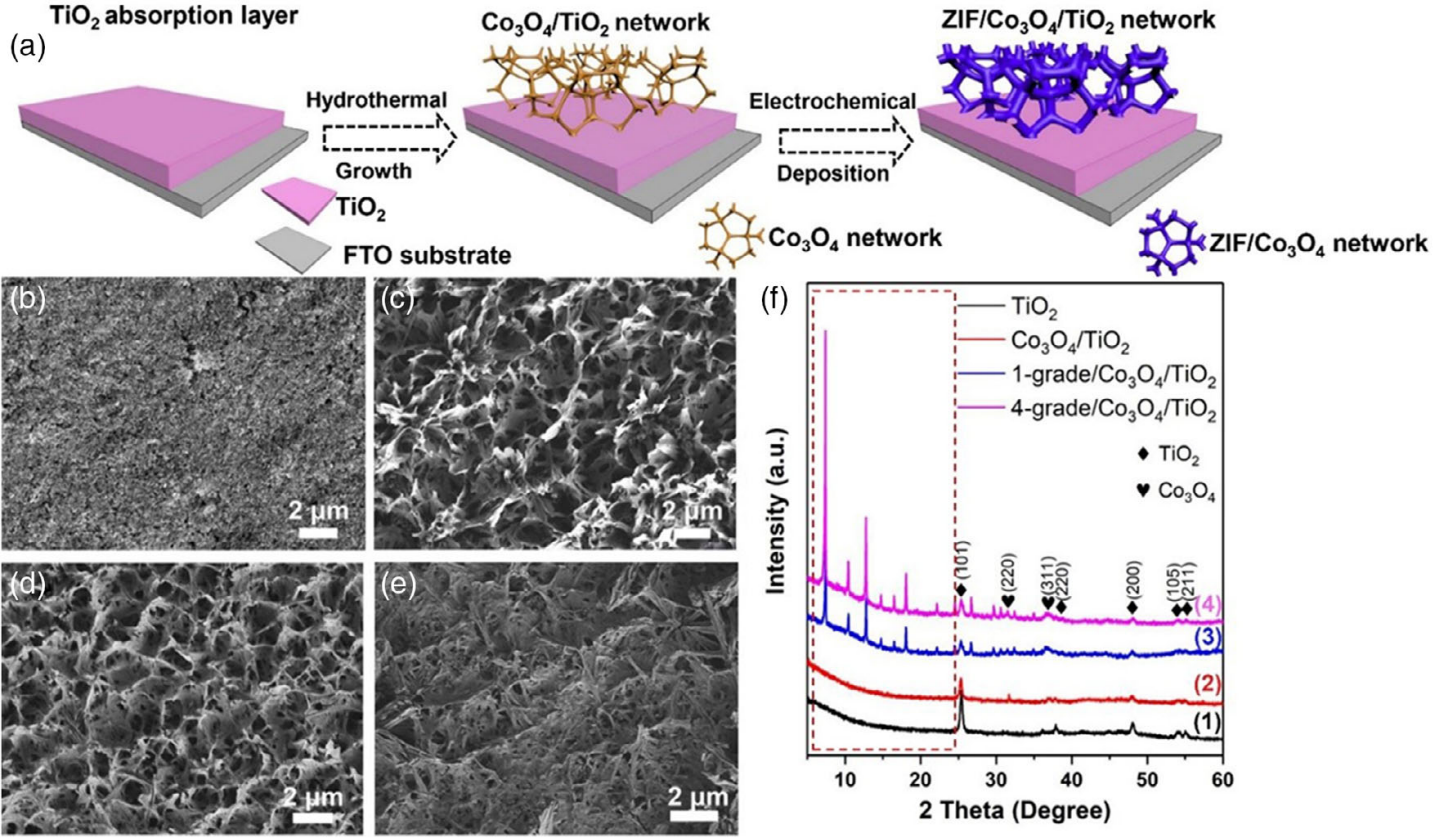
a) Schematic illustration of synthesis procedure of multi‐GHJ ZIF‐Co_*x*_Zn_1−*x*_/Co_3_O_4_/TiO_2_ network‐like photoanode. b–e) SEM images of TiO_2_ absorption layer, Co_3_O_4_/TiO_2_, 1‐grade/Co_3_O_4_/TiO_2_, and 4‐grade/Co_3_O_4_/TiO_2_ photoanodes. f) XRD patterns of TiO_2_ absorption layer, Co_3_O_4_/TiO_2_, 1‐grade/Co_3_O_4_/TiO_2_, and 4‐grade/Co_3_O_4_/TiO_2_ photoanodes.

As TiO_2_ is regarded as one of the most applied photoanode materials, it is applied here as a prototype semiconductor material to investigate the effect of MOF‐based multi‐GHJ architecture and structural design on carrier transport. In **Figure** **1**a, the typical synthesis process of ZIF‐Co_*x*_Zn_1−*x*_/Co_3_O_4_/TiO_2_ photoanode is shown. First, a TiO_2_ absorption layer is deposited onto the FTO substrate through a typical doctoral‐blade method. From Figure 1b and Figure S1a, Supporting Information, it can be seen that the TiO_2_ nanoparticles are uniformly covered on the FTO substrate. Then, through a hydrothermal method, a network‐like Co_3_O_4_ array is grown on the TiO_2_ layer. The interconnected structure of Co_3_O_4_ skeleton can be clearly seen (Figure 1c). From the transmission electron microscopy (TEM) images, the porous nanosheet structure of Co_3_O_4_ skeleton can be seen (Figure S2, Supporting Information). According to the cross‐section scanning electron microscopy (SEM), a thickness of 1.5 μm can be seen (Figure S1b, Supporting Information). Subsequently, through the facile multiple‐step electrodeposition method, ZIF‐Co_*x*_Zn_1−*x*_ with different gradients can be grown onto the Co_3_O_4_ skeleton with a rationally tailored Co/Zn ratio. Figure 1d shows the front‐view SEM image of 1‐grade/Co_3_O_4_/TiO_2_ sample; it is shown that the Co_3_O_4_ skeleton is thicker. Figure 1e shows the front‐view SEM image of 4‐grade/Co_3_O_4_/TiO_2_ sample, it is shown that the skeleton turns much thicker with better coverage, indicating that more ZIF‐Co_*x*_Zn_1−*x*_ is decorated onto the Co_3_O_4_ skeleton. As shown in Figure 1f, the X‐ray diffraction (XRD) patterns of TiO_2_, and Co_3_O_4_ can be clearly identified from the 1‐grade/Co_3_O_4_/TiO_2_ and 4‐grade/Co_3_O_4_/TiO_2_ samples (Line 3 and 4). The other peaks outlined in the dashed rectangle can be ascribed to the XRD patterns of ZIF‐Co_*x*_Zn_1−*x*_. In addition, it should be pointed out that 4‐grade/Co_3_O_4_/TiO_2_ shows a much stronger ZIF XRD intensity compared with 1‐grade/Co_3_O_4_/TiO_2_. It further confirms that through the multiple‐step electrochemical deposition process, the growth of ZIF‐Co_*x*_Zn_1−*x*_ can be efficiently tuned.

As the multi‐GHJ is composed of ZIF‐Co_*x*_Zn_1−*x*_ with different Zn/Co ratios, the corresponding components should be discussed. From the XRD patterns shown in Figure S3, Supporting Information, it is shown that with the Zn amount increasing, the crystal structure of ZIF‐Co_*x*_Zn_1−*x*_ shows hardly a change. But from the magnified XRD patterns, it is shown that with the Zn amount increasing, the characterization peaks show gradually negative‐shifted trends, indicating an increased lattice constant (Figure S3b, Supporting Information). This phenomenon suggests lattice distortion in ZIF‐Co, caused by the successful substitution of Co^3+^ by Zn^2+^.^[^ ^20^ ^]^ It is reported that the covalent radius of Zn^2+^ is larger than Co^3+^.^[^ ^20^ ^]^ Therefore, the doping of Zn^2+^ will lead to cell expansion of ZIF‐Co.

**Figure 2:**
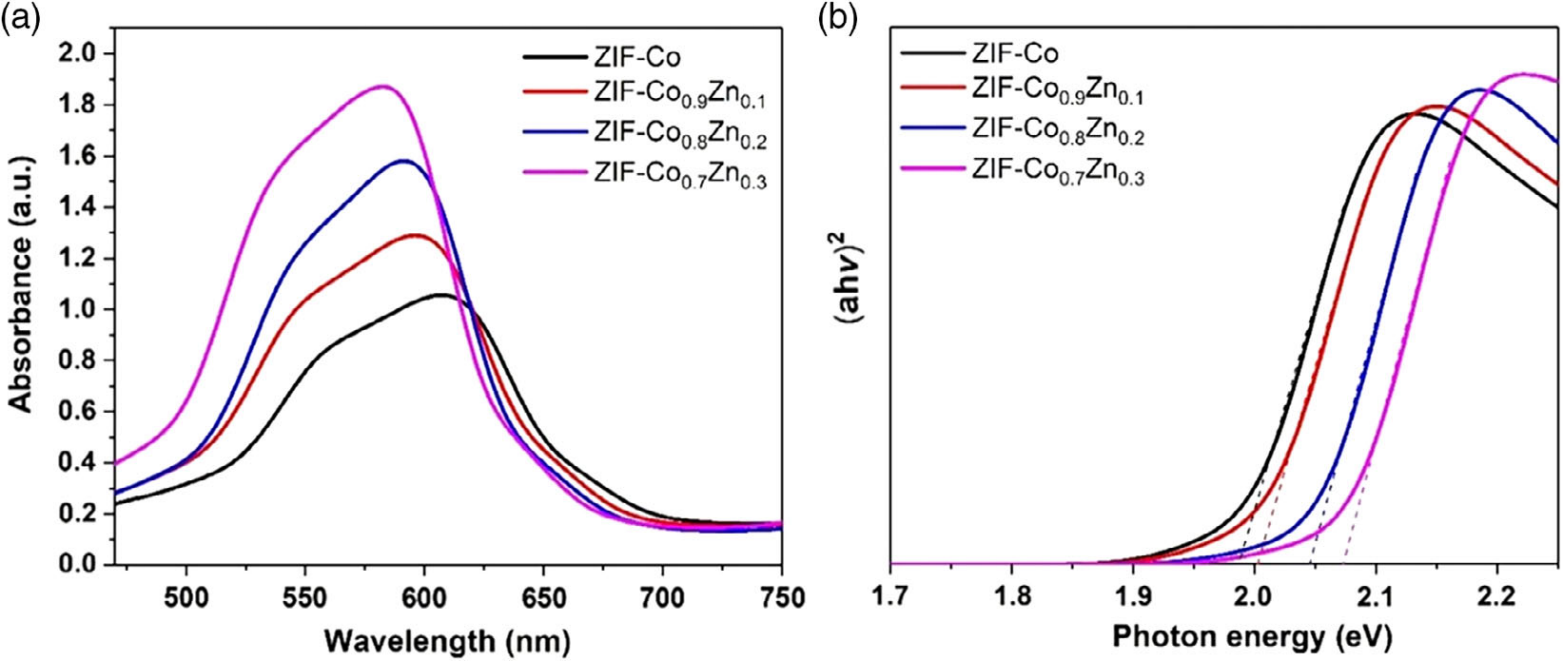
a) UV–vis absorbance spectra of ZIF‐Co, ZIF‐Co_0.9_Zn_0.1_, ZIF‐Co_0.8_Zn_0.2_, and ZIF‐Co_0.7_Zn_0.3_. b) Tauc plots of ZIF‐Co, ZIF‐Co_0.9_Zn_0.1_, ZIF‐Co_0.8_Zn_0.2_, and ZIF‐Co_0.7_Zn_0.3_ calculated from UV–vis absorbance spectra.

To investigate the effect of ZIF‐Co_*x*_Zn_1−*x*_ multi‐GHJ on the carrier separation performance, it is important to investigate the electron structure of ZIF‐Co_*x*_Zn_1−*x*_ (*x *= 1, 0.9, 0.8, 0.7) samples. Here, the energy band alignment of ZIF‐Co_*x*_Zn_1−*x*_ (*x *= 1, 0.9, 0.8, 0.7) samples is investigated through ultraviolet photoelectron spectroscopy (UPS). In Figure S4, Supporting Information, the UPS spectra of ZIF‐Co_*x*_Zn_1−*x*_ (*x* = 1.0, 0.9, 0.8, 0.7) are shown. By analyzing the UPS spectra,^[^ ^21^, ^22^ ^]^ the valence band maximum (VBM) of ZIF‐Co_*x*_Zn_1−*x*_ (*x* = 1.0, 0.9, 0.8, 0.7) is calculated to be −4.69, −5.08, −5.38, and −5.49 eV versus vacuum (vac), respectively. Moreover, through UV–vis absorbance spectra (**Figure** **2**a), the bandgaps of ZIF‐Co_*x*_Zn_1−*x*_ (*x *= 1, 0.9, 0.8, 0.7) samples are further investigated. According to the Tauc plot function

(1) $${\left(\alpha hv\right)}^{2}{=(hv‐E}_{g})$$

where *α* is the absorption coefficient, *h* is the Planck's constant, *v* is the photon's frequency, and *E*_g_ is the bandgap.^[^ ^23^, ^24^ ^]^ Hence, the bandgaps of ZIF‐Co_*x*_Zn_1−*x*_ (*x *= 1, 0.9, 0.8, 0.7) samples are calculated to be 1.98, 2.00, 2.05, and 2.08 eV (Figure 2b). Considering the Co/Zn ratio in the ZIF‐Co_*x*_Zn_1−*x*_ (*x *= 1, 0.9, 0.8, 0.7) samples, it can be seen that the bandgap shows a quasilinear relationship with the Zn amount increasing (Figure S5–S6, Supporting Information). Therefore, the conduction band (CB) positions of ZIF‐Co_*x*_Zn_1−*x*_ (*x* = 1.0, 0.9, 0.8, 0.7) are calculated to be −2.61, −3.08, −3.38, and −3.51 eV versus vac. This result confirms that by rationally tailoring the Co/Zn ratio, ZIF‐Co_*x*_Zn_1−*x*_ with a continuously adjustable energy band structure can be obtained. Therefore, it can be confirmed that a multi‐GHJ can be constructed by depositing the ZIF‐Co_*x*_Zn_1*−x*_ (*x *= 1.0, 0.9, 0.8, 0.7) samples layer by layer (Figure S7, Supporting Information).

Here, the PL spectra of 1‐grade, 2‐grade, 3‐grade, and 4‐grade heterojunctions are shown in Figure S8, Supporting Information. It is shown that by constructing multi‐GHJ, the PL intensity shows a decreasing trend, indicating that the multi‐GHJ can indeed suppress the recombination of photoinduced carriers.^[^ ^25^, ^26^ ^]^

**Figure 3:**
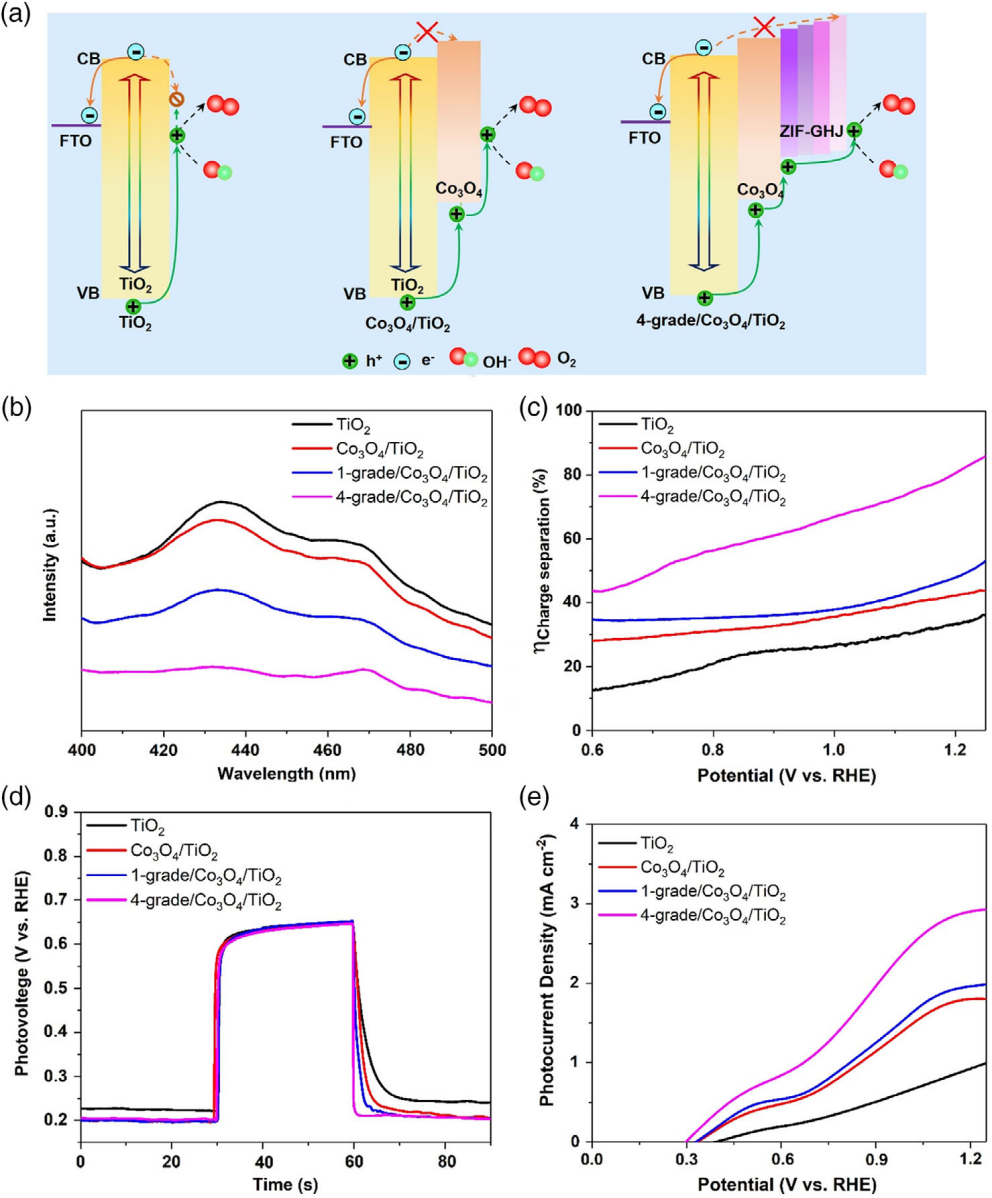
a) Energy band alignments and photoinduced electron/hole pairs transport processes for pristine TiO_2_, Co_3_O_4_/TiO_2_, and multi‐GHJ architecture Co_3_O_4_/TiO_2_ photoanode. b) PL spectra, c) charge separation efficiency plots, d) Photovoltage–decay plots, and e) LSV curves of pristine TiO_2_, Co_3_O_4_/TiO_2_, 1‐grade/Co_3_O_4_/TiO_2_, and 4‐grade/Co_3_O_4_/TiO_2_ samples.

To further investigate the effect of multi‐GHJ on carrier separation and PEC catalytic performance, the multi‐GHJ architecture is deposited onto the top of the Co_3_O_4_/TiO_2_ photoanode. When a semiconductor photoanode is excited by the incident light, ideally, photoinduced holes will be transported to the electrode/electrolyte interface, whereas the photoinduced electrons are collected at the FTO substrate. However, in practical cases, photogenerated electrons will not only be transported to the FTO, but some electrons are also back transported to the electrode/electrolyte interface, which will be trapped by unreacted photogenerated holes and lead to severe carrier recombination, thereby reducing the PEC catalytic performance.^[^ ^7^ ^]^ **Figure** **3**a shows the energy band structure among TiO_2_, Co_3_O_4_/TiO_2_, and 4‐grade/Co_3_O_4_/TiO_2_. For the pristine TiO_2_ layer, when the photoinduced carrier is excited, the photoinduced electrons are supposed to be transported to FTO. However, some electrons will back transfer and recombine with photoinduced holes, leading to serious carrier loss. For the Co_3_O_4_/TiO_2_ photoanode, according to a previously reported work, a heterojunction can be formed at the Co_3_O_4_/TiO_2_ interface, which can prevent the back‐transfer of electrons and extract the photoinduced holes from the VB of TiO_2_.^[^ ^27^ ^]^ It was reported that both the CB and valence band positions of Co_3_O_4_ are lower than that of ZIF‐Co.^[^ ^28^ ^]^ Therefore, after the deposition of multi‐GHJ, the back‐transfer process of electrons can be further suppressed. With the negatively shifted valance band position of ZIF‐Co_*x*_Zn_1−*x*_ (*x *= 1, 0.9, 0.8, 0.7), the multi‐GHJ can act as the hole transport channel and provide an extra hole extraction driving force. Thanks to the additional driving force provided by the multi‐GHJ, the electron/hole separation kinetics can be greatly improved, which will lead to an improved PEC catalytic performance.

To demonstrate the aforementioned carrier migration behavior, the PL spectra of TiO_2_, Co_3_O_4_/TiO_2_, 1‐grade/Co_3_O_4_/TiO_2_, and 4‐grade/Co_3_O_4_/TiO_2_ are shown in Figure 3b. The pristine TiO_2_ shows obviously a strong PL intensity, attributing to its unavoidable intense photoinduced carrier recombination process.^[^ ^29^ ^]^ For the Co_3_O_4_/TiO_2_ sample, the PL intensity shows a slight decline, due to the carrier separation process caused by the Co_3_O_4_/TiO_2_ heterojunction. In comparison, 4‐grade/Co_3_O_4_/TiO_2_ shows the weakest PL peak intensity, indicating the best carrier separation performance.

To further illustrate the correlation between carrier separation and PEC catalytic performance, a typical electrochemical method is applied to evaluate the carrier separation efficiency of the pristine TiO_2_, Co_3_O_4_/TiO_2_, 1‐grade/Co_3_O_4_/TiO_2_, and 4‐grade/ Co_3_O_4_/TiO_2_ samples and is shown in Figure 3c.^[^ ^26^, ^30^ ^]^ It is shown that, compared with the pristine TiO_2_, the Co_3_O_4_/TiO_2_ sample shows enhanced charge separation, due to the Co_3_O_4_/TiO_2_ heterojunction. After 1‐grade ZIF‐Co is loaded, the efficiency shows a further enhancement. Then, by rationally tailoring to the 4‐grade photoanode, a greatly improved carrier separation efficiency is shown. At 1.23 V versus reversible hydrogen electrode (RHE), a maximum $\eta_{\text{charge separation}}$ of 83.8% can be obtained. This confirms that the carrier separation behavior can indeed be improved by the additional driving force brought by the multi‐GHJ architecture.

Recently, photovoltage–time (*V–t*) decay plots have been considered as an efficient way to characterize surface carrier trapping in photoanode materials.^[^ ^31^, ^32^ ^]^ Here, the *V–t* plots of pristine TiO_2_, Co_3_O_4_/TiO_2_, 1‐grade/Co_3_O_4_/TiO_2_, and 4‐grade/Co_3_O_4_/TiO_2_ samples are collected under the three‐electrode condition (Figure 3d). According to Li and coworkers,^[^ ^33^ ^]^ as recorded, *V–t* plots are fitted by the biexponential function with two time constants.

(2) $${y(t)=A}_{0}+A_{1}e^{-t/\tau 1}+A_{2}e^{-t/\tau 2}$$

(3) $$\tau_{m}=\frac{\tau_{1}\times \tau_{2}}{\tau_{1}+\tau_{2}}$$

Among which, *τ*_m_ is the harmonic mean of the lifetime and the total half lifetime can be calculated by

(4) $$\tau_{h}={\log(2\times \tau }_{m})$$

According to Equation ((3), (4), (5)), the total half lifetime can be calculated to be 0.65, 0.49, 0.47, and 0.28 s for TiO_2_, Co_3_O_4_/TiO_2_, 1‐grade/Co_3_O_4_/TiO_2_, and 4‐grade/Co_3_O_4_/TiO_2_ samples, respectively. Due to the heterojunction between Co_3_O_4_ and TiO_2_, the Co_3_O_4_/TiO_2_ sample shows more rapid decay compared with the pristine TiO_2_, which is in accordance with the charge separation efficiency results. Moreover, for the 4‐grade/Co_3_O_4_/TiO_2_ sample, the fastest decay kinetics is recorded. This result confirms that the construction of multi‐GHJ hole extraction layers can indeed improve the carrier separation capability of the photoanode materials.

The morphology of the photoanode also has great influence on its carrier transport performance.^[^ ^34^ ^]^ Here, planar structured 4‐grade/Co_3_O_4_/TiO_2_ samples (p‐4‐grade/Co_3_O_4_/TiO_2_) are also synthesized. The carrier separation efficiency and *V–t* plot of p‐4‐grade/Co_3_O_4_/TiO_2_ and the network‐like 4‐grade/Co_3_O_4_/TiO_2_ (n‐4‐grade/Co_3_O_4_/TiO_2_) samples are shown in Figure S9, Supporting Information. It is shown that the n‐4‐grade/Co_3_O_4_/TiO_2_ sample achieves better carrier separation and faster decay kinetics. It confirms that due to the larger surface area of the network‐like Co_3_O_4_ skeleton compared with the planar‐structured Co_3_O_4_, better interfacial carrier transport performance can be achieved.

The detailed carrier transport direction is further analyzed by X‐ray photoelectron spectroscopy (XPS) (Figure S10, Supporting Information). First, the O 1s spectra of pristine TiO_2_, Co_3_O_4_, Co_3_O_4_/TiO_2_, 4‐grade/TiO_2_, 4‐grade/Co_3_O_4_/TiO_2_, and 4‐grade/Co_3_O_4_ samples are tested. It can be seen that compared with pristine Co_3_O_4_,^[^ ^35^ ^]^ due to the modification of the MOF‐based multi‐GHJ, the Co–O peak shows a slightly negative shift, indicating the increased electron density of Co_3_O_4_. According to the work reported by Shao and coworkers,^[^ ^26^, ^36^, ^37^ ^]^ this result is caused by the electron transport from the MOF‐based multi‐GHJ to Co_3_O_4_. Furthermore, compared with pristine TiO_2_,^[^ ^26^ ^]^ the Ti–O peaks of Co_3_O_4_/TiO_2_ and 4‐grade/TiO_2_ show a negative shift trend. It indicates that the electrons are transported from Co_3_O_4_ and the MOF‐based multi‐GHJ to the pristine TiO_2_, which also means that the holes are transported from TiO_2_ to Co_3_O_4_ and MOF‐based multi‐GHJ. Within the 4‐grade/Co_3_O_4_/TiO_2_ sample, a more obvious negative shift trend of the Ti–O peak can be seen. Considering all the carrier transport directions discussed earlier, we can confirm that the 4‐grade/Co_3_O_4_/TiO_2_ sample can realize a carrier transport direction where the electrons transport from the MOF‐based multi‐GHJ to Co_3_O_4_ and then to TiO_2_. Also, the holes are transported from TiO_2_ to Co_3_O_4_ and then the MOF‐based multi‐GHJ, which is consistent with our carrier transport model (Figure 3a). Moreover, the Ti 2p XPS spectra of pristine TiO_2_, Co_3_O_4_/TiO_2_, 4‐grade/TiO_2_, and 4‐grade/Co_3_O_4_/TiO_2_ are further tested and discussed. Compared with pristine TiO_2_,^[^ ^26^ ^]^ it can be seen that both the Ti 2p_1/2_ and Ti 2p_3/2_ peaks of Co_3_O_4_/TiO_2_ and 4‐grade/TiO_2_ show a slightly negative shift, which should be attributed to the electron density increase of TiO_2_. It indicates that the electrons are transported from Co_3_O_4_ and the MOF‐based multi‐GHJ to the pristine TiO_2_, which also means that the holes are transported from TiO_2_ to Co_3_O_4_ and MOF‐based multi‐GHJ. Further, the 4‐grade/Co_3_O_4_/TiO_2_ sample shows a more obvious negative shift trend. Considering both Ti 2p and O 1s spectra, it suggests that within the 4‐grade/Co_3_O_4_/TiO_2_ sample, the electrons transported from the MOF‐based multi‐GHJ to Co_3_O_4_ and then to TiO_2_, with the holes transported in the opposite direction. Therefore, combing the XPS results and the theoretical and experimental results tested under light illumination, it can be confirmed that the as‐constructed heterojunction photoanode can indeed improve the system's carrier separation performance.

To evaluate the effect of this facilitated carrier separation performance brought by multi‐GHJ and the unique morphology of photoanode, the linear sweep voltammogram (LSV) curves of all samples are shown in Figure 3e. It can be seen that, due to the poor light‐harvesting capability and severe carrier recombination of the pristine TiO_2_, the corresponding photocurrent density is quite low. By modifying TiO_2_ with Co_3_O_4_, the photocurrent density exhibits an obvious enhancement. This may be attributed to the improved carrier transport performance induced by the Co_3_O_4_/TiO_2_ heterojunction. As for the 1‐grade/Co_3_O_4_/TiO_2_ sample, a further enhanced photocurrent density can be evidenced. For the 4‐grade/Co_3_O_4_/TiO_2_ sample, the best PEC water oxidation performance of 2.91 mA cm^−2^ at 1.23 V versus RHE is achieved. From the LSV curves of the photoanode with different gradients (Figure S11, Supporting Information), it is shown that the 4‐grade/Co_3_O_4_/TiO_2_ sample also achieves the best performance. Considering that the 4‐grade/Co_3_O_4_/TiO_2_ sample achieves the lowest PL peak intensity, the highest charge separation efficiency, and the fastest photovoltage decay rate, it can be concluded that, by constructing the MOF‐based multi‐GHJ, the PEC water oxidation performance can be significantly enhanced. The morphology influence of the Co_3_O_4_ carrier transport layer is also investigated, and the LSV plots of p‐4‐grade/Co_3_O_4_/TiO_2_ and n‐4‐grade/Co_3_O_4_/TiO_2_ are shown in Figure S12, Supporting Information. It is shown that n‐4‐grade/Co_3_O_4_/TiO_2_ achieves a better PEC performance. This result can be ascribed to the unique network‐like structured photoanode, which will not only lead to a better carrier separation process, but also improved electrode/electrolyte interfacial mass transport capability.^[^ ^38^ ^]^ To further verify the PEC enhancement achieved by 4‐grade/Co_3_O_4_/TiO_2_, the recorded photocurrent is renormalized by the electrochemical active surface area (ECSA). As shown in Figure S13–S14, Supporting Information, it is shown that the 4‐grade/Co_3_O_4_/TiO_2_ photoanode achieves the best PEC performance.

The incident photon‐to‐current conversion efficiency (IPCE) determined by the optical response and carrier separation^[^ ^39^ ^]^ are shown in Figure S15, Supporting Information, to further confirm the enhanced PEC performance. After the growth of the network‐like Co_3_O_4_ skeleton, IPCE is enhanced. This can be attributed to three aspects. First, due to the heterojunction between Co_3_O_4_ and TiO_2_, its carrier separation capability can be improved. Second, compared with TiO_2_, Co_3_O_4_/TiO_2_ shows a larger optical response range, due to the narrow‐bandgap property of Co_3_O_4_. Third, as the network‐like Co_3_O_4_ skeleton will lead to the multilight scattering process (Figure S16, Supporting Information), the light capture capability of the photoanode material is also enhanced.^[^ ^40^, ^41^ ^]^ Due to the multilight scattering process provided by the network‐like Co_3_O_4_ skeleton, the traveling length of the incident light is greatly enhanced, which therefore leads to enhanced optical harvesting performance. After decorating the multi‐GHJ, IPCE shows further improvement. This can be assigned to the multi‐GHJ‐induced better carrier separation kinetics. Moreover, due to the narrow‐bandgap character of ZIF‐Co_*x*_Zn_1−*x*_, an extra peak appears in the wavelength range of 500–650 nm, which is in accordance with the UV–vis absorbance spectra and electron flux spectra (Figure S17, Supporting Information). This can also contribute to the enhanced PEC performance. Therefore, the greatly improved PEC water oxidation performance achieved by the network‐like 4‐grade/Co_3_O_4_/TiO_2_ can be attributed to the synergistic effect of the unique photoanode component, morphology, and the rationally designed multi‐GHJ architecture.

**Figure 4:**
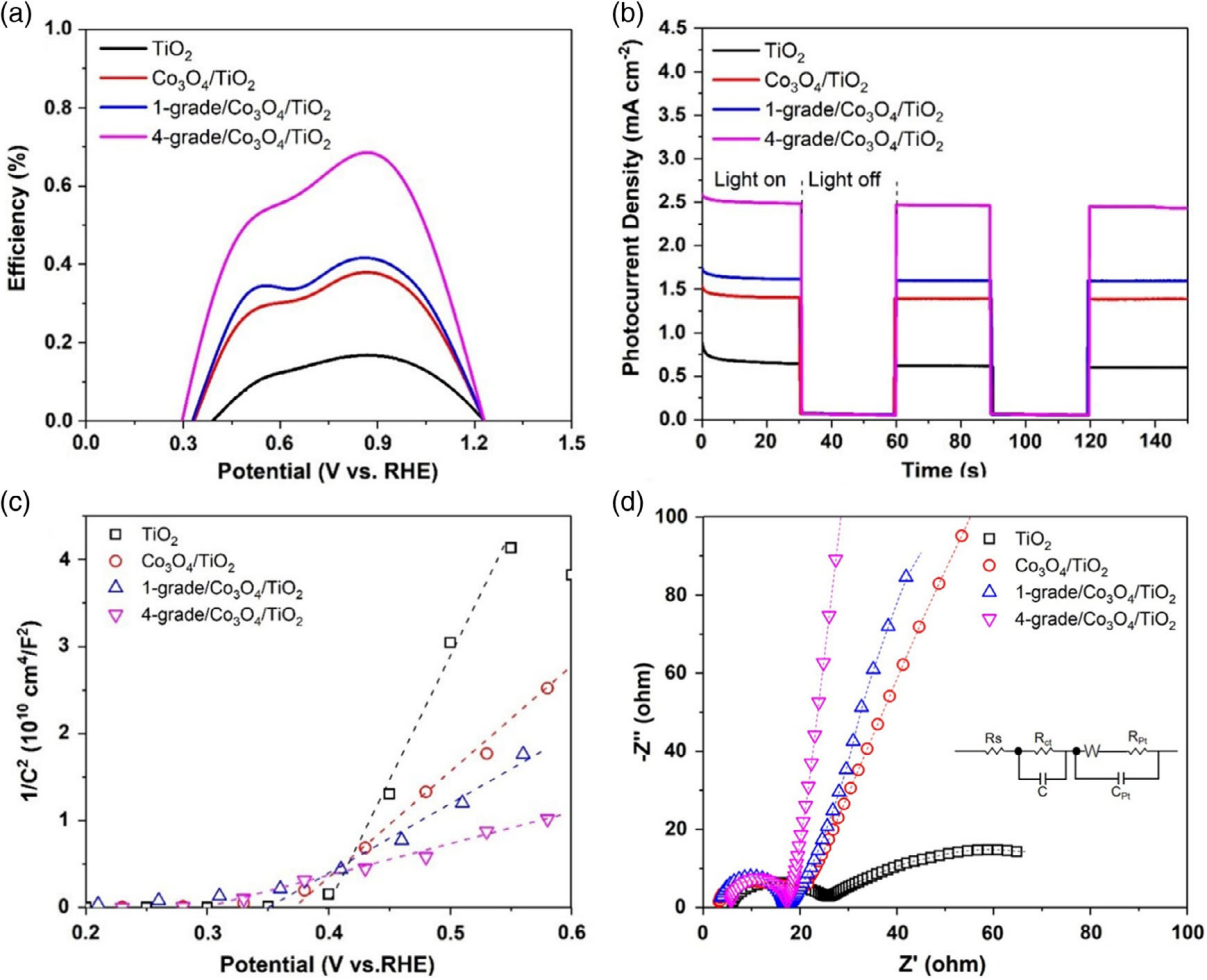
a) ABPE plots, b) chronoamperometric *I–t* curves at a bias potential of 1 V under chopped illumination, c) Mott–Schottky plots, and d) electrochemical impedance spectra measured at a bias potential of 1.0 V for TiO_2_, Co_3_O_4_/TiO_2_, 1‐grade/Co_3_O_4_/TiO_2_, and 4‐grade/Co_3_O_4_/TiO_2_ samples.

According to the LSV plots, the applied‐bias photon‐to‐current conversion efficiency (ABPE) is shown in **Figure** **4**a. It is shown that the 4‐grade/Co_3_O_4_/TiO_2_ sample achieves the largest photoelectric conversion efficiency (PCE) of about 0.7% at the bias potential of around 0.86 V versus RHE. Under the same bias potential, the PCE of Co_3_O_4_/TiO_2_ is about 0.38%, compared with that of the pristine TiO_2_ for 0.16%. It demonstrates that the PCE can be efficiently improved by the introduction of the MOF‐based multi‐GHJ architecture, which sequentially leads to a better PEC catalytic performance.

The optical response performance of the as‐synthesized photoanode materials is further investigated by the chronoamperometric *I–t* curves under chopped illumination. As shown in Figure 4b, it can be seen that all samples show rapid photocurrent change with the incident light on and off circulation. With the light on, the photocurrent densities show a decreasing tendency of 4‐grade/Co_3_O_4_/TiO_2_ > 1‐grade/Co_3_O_4_/TiO_2_ > Co_3_O_4_/TiO_2_ > TiO_2_, which aligns well with the LSV results. As illustrated before, due to the multi‐GHJ‐induced electric field, the recombination of photoinduced electron/hole pairs can be greatly suppressed. More photoinduced electron–hole pairs can be generated with a better light capture capability, and more photoinduced holes can reach the electrode/electrolyte interface, leading to better water oxidation catalytic performance.

In Figure 4c, the Mott–Schottky plots of pristine TiO_2_, Co_3_O_4_/TiO_2_, 1‐grade/Co_3_O_4_/TiO_2_, and 4‐grade/Co_3_O_4_/TiO_2_ samples show the semiconductor properties of the as‐synthesized photoanode materials. It is shown that all samples have positive slopes, indicating the n‐type properties of as‐synthesized materials.^[^ ^42^ ^]^ As previously reported, the carrier density within the photoanode materials can be determined by the Mott–Schottky plots, which is inversely proportional to the slope.^[^ ^26^ ^]^ A decreasing trend of 4‐grade/Co_3_O_4_/TiO_2_ > 1‐grade/Co_3_O_4_/TiO_2_ > Co_3_O_4_/TiO_2_ > TiO_2_ is evidenced. This result corroborates well with the recorded charge separation efficiency and PL plots and proves that by introducing the MOF‐based multi‐GHJ architecture, the recombination of photoinduced carriers can be greatly suppressed and more effective holes can be retained to participate in the water oxidation reaction. By extrapolating the *X*‐interception of the linear region, it is shown that the 4‐grade/Co_3_O_4_/TiO_2_ sample achieves the most negatively shifted flat‐band potential. Such a tendency also indicates a higher concentration in the 4‐grade/Co_3_O_4_/TiO_2_ sample, which leads to more efficient charge collection on the FTO current collector.^[^ ^26^ ^]^ This is consistent with the carrier transport model constructed earlier (Figure 3a).

Electrochemical impedance spectroscopy (EIS) is also applied to study the carrier transport behavior of the designed photoanode materials. The equivalent circuit model of the synthesized photoanodes is shown in the inset of Figure 4d. According to previous report,^[^ ^43^ ^]^ *R*_s_, *R*_ct_, *C*, and *W* are assigned to the series resistance, charge transfer resistance, space–charge region capacitance, and Warburg impedance, respectively. Meanwhile, *R*_Pt_ and *C*_Pt_ refer to the resistance and capacitance at the Pt cathode. As shown in Figure 4d, the EIS plots can be divided into two parts. According to the work reported before, the small semicircle at the high frequency refers to the charge transfer resistance (*R*_ct_) and the straight line at the low‐frequency region refers to mass transfer resistance.^[^ ^43^, ^44^, ^45^ ^]^ From Figure 4d, it is shown that the largest *R*_ct_ (17.2 Ω) and mass transport resistance (60.5 Ω) are achieved by the pristine TiO_2_, indicating the unfavorable photoanode/electrolyte charge transfer and mass transport. In comparison, the 4‐grade/Co_3_O_4_/TiO_2_ sample achieves the smallest charge transfer resistance (12.1 Ω) and mass transfer resistance (30.4 Ω), which should be attributed to the synergistic effect of the MOF‐based multi‐GHJ, leading to better charge transport kinetics and skeleton structure photoanode‐induced mass transport.^[^ ^26^, ^43^, ^46^ ^]^ The EIS plots of p‐4‐grade/Co_3_O_4_/TiO_2_ and n‐4‐grade/Co_3_O_4_/TiO_2_ are shown in Figure S18, Supporting Information. Compared with p‐4‐grade/Co_3_O_4_/TiO_2_, n‐4‐grade/Co_3_O_4_/TiO_2_ also achieves better charge migration and mass transport capability. This result further confirms that the system PEC activity can be enhanced due to the large surface area of the network‐like Co_3_O_4_ skeleton.

**Figure 5:**
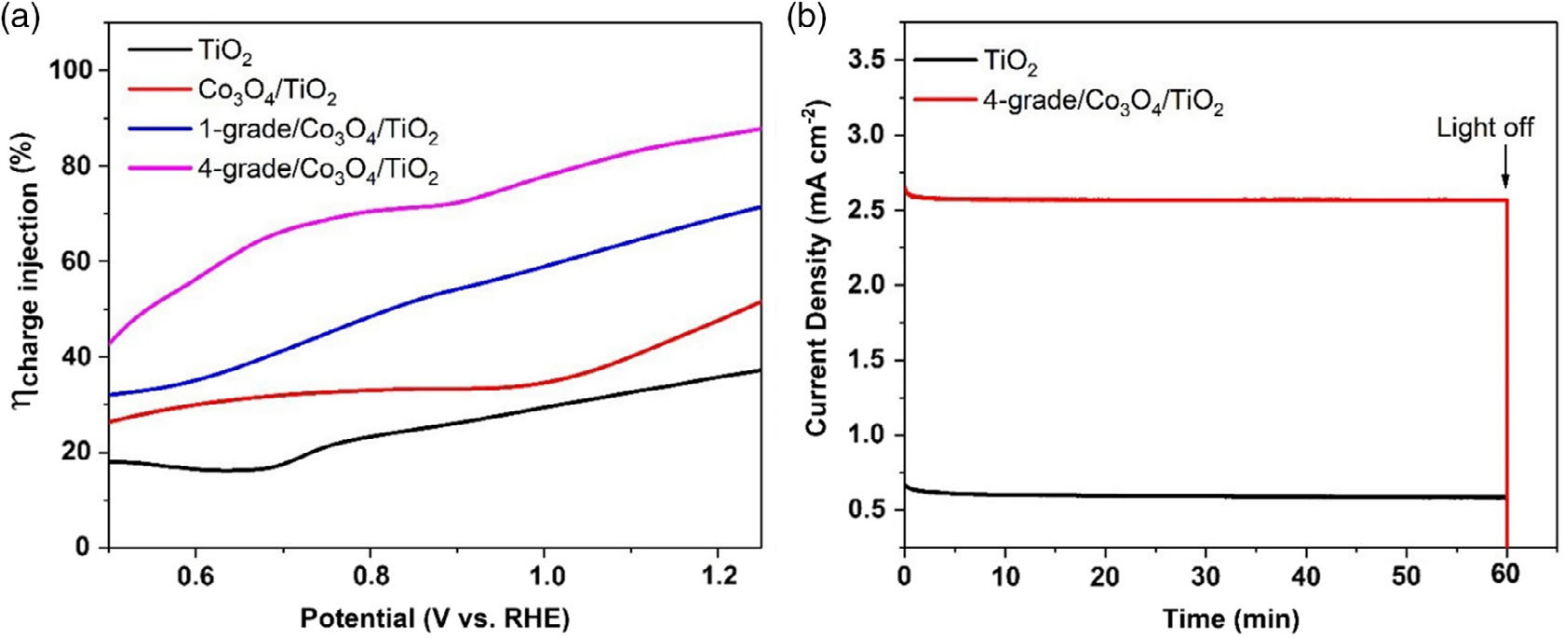
a) Charge injection efficiency versus potential curves of TiO_2_, Co_3_O_4_/TiO_2_, 1‐grade/Co_3_O_4_/TiO_2_, and 4‐grade/Co_3_O_4_/TiO_2_ samples. b) Chronoamperometric *I–t* curves of TiO_2_ and 4‐grade/Co_3_O_4_/TiO_2_ at a bias potential of 1.0 V versus RHE.

To further investigate the effect of the MOF‐based multi‐GHJ‐induced unique photoanode/electrolyte interfacial carrier migration behavior on the enhanced PEC water oxidation performance, the interfacial charge injection process is discussed.^[^ ^26^, ^30^ ^]^ The charge injection efficiency is obtained from the photocurrent density collected from H_2_O and Na_2_SO_3_ solution. As shown in **Figure** **5**a, compared with the pristine TiO_2_ (28.4%, 1.23 V vs RHE), Co_3_O_4_/TiO_2_, 1‐grade/Co_3_O_4_/TiO_2_, and 4‐grade/Co_3_O_4_/TiO_2_ samples achieve obviously enhanced charge separation efficiency. This enhancement can be attributed to the cocatalyst effect induced by Co_3_O_4_ and ZIF‐Co. It is reported earlier due to the cobalt‐rich structure of ZIF‐Co, which is a highly efficient water oxidation cocatalyst.^[^ ^47^, ^48^ ^]^ For the 4‐grade/Co_3_O_4_/TiO_2_ sample, the photoinduced hole can be efficiently consumed by the OH^−^ from the electrolyte through the ZIF‐Co_*x*_Zn_1−*x*_‐based photoanode interface. It is worth mentioning that such a facilitated hole consumption process will also lead to better carrier separation capability as the surficial *e* ^−^/*h* ^+^ trapping processes can be efficiently suppressed. Therefore, the concurrent effect of energy band engineering and the cocatalyst affords an outstanding carrier migration capability of 73.3% for the constructed 4‐grade/Co_3_O_4_/TiO_2_ photoanode, which is determined by *η*_charge separation_ × *η*_charge injection_ (*η*_s_ × *η*_i_). To the best of our knowledge, due to the synergy effect of the MOF‐based multi‐GHJ‐induced carrier separation enhancement and ZIF‐Co‐induced charge injection capability, greatly enhanced carrier separation and charge migration capability are achieved, which are comparable or even better to some recently reported works on PEC water oxidation. For example, the highest photocurrent density of BiVO_4_‐N/C‐CoPOM photoanode is 3.30 mA cm^−2^, whereas a carrier migration efficiency of 58.9% is obtained.^[^ ^49^ ^]^ A Lu_2_O_3_‐modified BiVO_4_ photoanode is constructed by Liu and coworkers, with the highest photocurrent density of 1.72 mA cm^−2^ and carrier migration efficiency of 36.2%.^[^ ^50^ ^]^ More comparisons are shown in Table S1, Supporting Information. Therefore, compared with the pristine semiconductors, the constructed multi‐GHJ architecture photoanode indeed achieves an outstanding PEC performance and carrier migration behavior.

The stability of the as‐designed photoanode material is shown in Figure 5b. During the 1 h *I–t* testing, the photocurrent density of the pristine TiO_2_ shows some decrease of about 9%. It may be attributed to the photocorrosion caused by the sluggish water oxidation kinetics of TiO_2_.^[^ ^51^ ^]^ The 4‐grade/Co_3_O_4_/TiO_2_ sample shows a much better photostability (a decrease of about 3%). This result indicates that the modification of Co_3_O_4_ and multi‐GHJ can effectively enhance the photoanode stability by accelerating the electrode/electrolyte interfacial hole consumption. The survey XPS of the fresh and used photoanode are detected. It is shown that for the fresh photoanode, the elements of Ti, O, Co, Zn, C, and N are detected (Figure S20, Supporting Information). After the cycling reaction, all elements can still be detected, which confirms that no‐metal ion dissolution occurs during the reaction. Moreover, in Figure S21, Supporting Information, the XRD pattern of the 4‐grade/Co_3_O_4_/TiO_2_ sample after the reaction shows that the phase component shows no obvious change after the reaction, which confirms the good stability of the as‐synthesized photoanode material.

**Figure 6:**
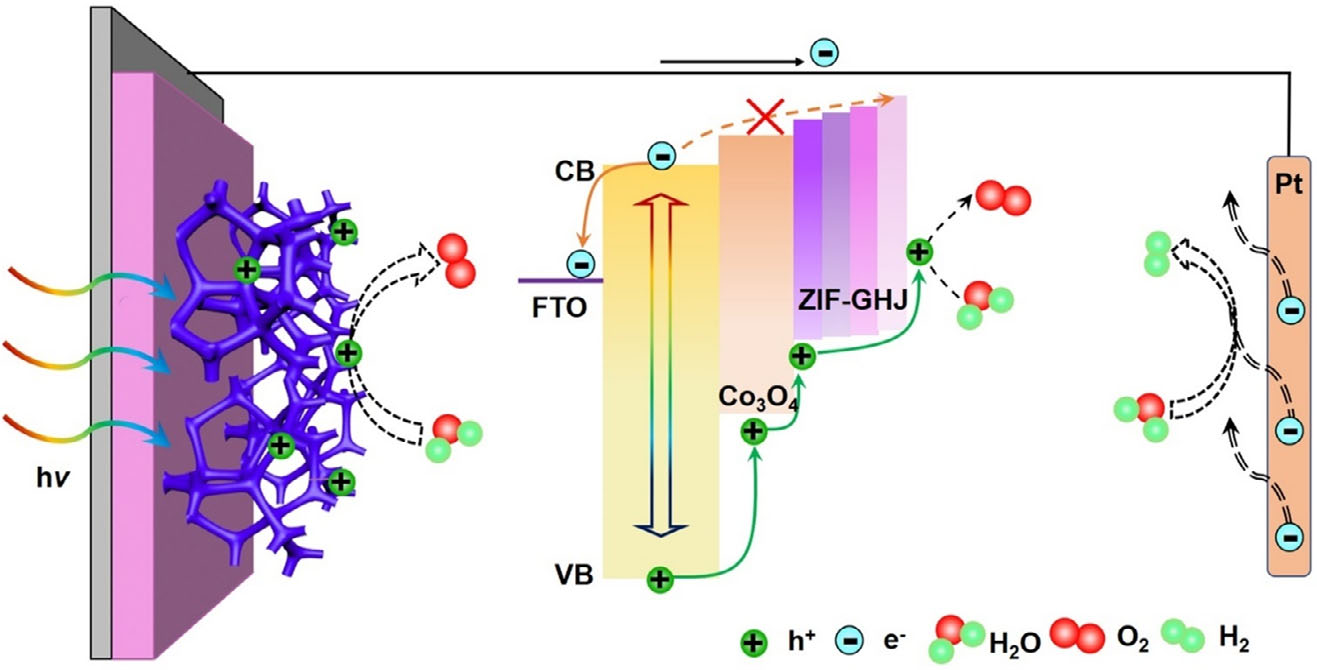
Schematic illustrations of the light harvesting and carrier separation mechanisms in the multi‐GHJ/Co_3_O_4_/TiO_2_ system.

According to the aforementioned discussion, a possible reaction process schematic based on the multi‐GHJ architecture photoanode is shown in **Figure** **6**. First, by constructing ZIF‐Co_*x*_Zn_1−*x*_ multi‐GHJ as the hole extraction channel, the photoinduced charge carrier recombination of the photoanode can be efficiently suppressed. Second, acting as the cocatalyst, ZIF‐Co will result in an obviously facilitated consumption of holes and oxidize the OH^−^ from the electrolyte into O_2_, which will further suppress the surficial carrier trapping recombination. Third, due to the uniquely designed Co_3_O_4_ skeleton, the network‐like character leads to greatly improved carrier migration and mass transport performance. Meanwhile, due to the multilight scattering effect induced by the skeleton, better light capture performance can be evidenced. Therefore, through rational structural design and energy band engineering, simultaneous enhancements on photoinduced carrier excitation separation and interfacial water oxidation kinetics can be evidenced with boosted PEC water oxidation performance.

## 3 Conclusion

In summary, a brand‐new MOF‐based multi‐GHJ architecture‐modified ZIF‐Co_*x*_Zn_1−*x*_/Co_3_O_4_/TiO_2_ photoanode is synthesized for the first time through a facile hydrothermal electrochemical deposition method. It is demonstrated that by rationally adjusting the Co/Zn ratio, the energy band structure of ZIF‐Co_*x*_Zn_1−*x*_ can be continuously tuned. By further reasonable structural design and energy band engineering, the MOF‐based multi‐GHJ architecture‐modified photoanode exhibits a significantly improved carrier separation–extraction capability and thus enhanced PEC performance. Remarkably, the 4‐grade/Co_3_O_4_/TiO_2_ electrode shows the best PEC water oxidation performance (2.91 mA cm^−2^ at 1.23 V vs RHE), with charge migration performance of 73.3%. This could be attributed to the following three factors. First, acting as the photoinduced hole transport channels, the multi‐GHJ architecture can greatly facilitate the efficient separation and extraction of photoinduced carriers, which is the primary advantage of this novel photoelectrode structure. Second, due to the cobalt‐rich character, ZIF‐Co can act as a cocatalyst and lead to significantly improved interfacial water oxidation performance, which further suppresses the surficial carrier trapping recombination process. Third, compared with the conventional planar‐structured photoanode, the unique network‐like Co_3_O_4_ skeleton provides better mass transport and light capture capability. This work first reports a novel MOF‐based multi‐GHJ architecture‐modified photoanode with outstanding charge carrier separation extraction efficiency, which shows great potential for the construction of high‐performance optoelectrical applications, such as photoelectrocatalytic applications, solar energy conversion, biological imaging fields, etc.

## 4 Experimental Section

4.1

4.1.1

##### Preparation of TiO_2_ Absorption Layer

The TiO_2_ absorption layer was synthesized through a facile doctoral‐blade method. The paste fabrication method was as follows: 0.1 g Anatase TiO_2_ power was dispersed in methanol by sonication. Then, 0.01 g polyethylene glycol was added for a further sonication process. Then, 0.5 ml paste was dropped on the FTO glass for a typical doctoral‐blade procedure. Finally, the substrate was annealed in air at 500 °C for 60 min to remove the organic compound and crystallize the photoanode materials.

##### Preparation of Co_3_O_4_ Network‐Like Array

The Co_3_O_4_ network‐like array was deposited onto the fluorine‐doped tin oxide (FTO) substrate as described elsewhere.^[^ ^52^, ^53^ ^]^ Then, a precursor solution was prepared according to a typical method as below; cobalt acetate and urea were mixed thoroughly in 100 ml deionized (DI) water. Then, the FTO substrate was suspended upside down in the aqueous solution and heated at 120 °C for 4 h. Thereafter, a layered cobalt carbonate hydroxide (LCCH) array was obtained. To convert LCCH into Co_3_O_4_, the sample was annealed in air at 500 °C for 0.5 h. For comparison, a planar‐structured Co_3_O_4_ layer was also deposited onto the TiO_2_ absorption layer by the doctoral‐blade method and annealed in air at 500 °C for 0.5 h.

##### Preparing ZIF‐Co_x_Zn_1−x_/Co_3_O_4_/TiO_2_

To prepare ZIF‐Co_*x*_Zn_1−*x*_/Co_3_O_4_/TiO_2_, a series of graded electrolytes were prepared. For example, a mixture (0.1 M) of cobalt acetate and zinc acetate (mole ratio Co/Zn = 9:1) and 0.15 M 2‐methylimidazole were quickly added into a mixed solution containing 45 ml methanol and 5 ml DI water and stirred vigorously for 30 s.^[^ ^54^ ^]^ Then, a three‐electrode system was applied, with the Co_3_O_4_/TiO_2_ as the working electrode. The depositing potential was −5.0 V versus Ag/AgCl. The final product was further annealed at 150 °C for 1 h to crystalize the photoanode materials. To synthesize the multi‐GHJ‐structured ZIF‐Co_*x*_Zn_1−*x*_/Co_3_O_4_/TiO_2_, the electrolyte was changed by tailoring the Co/Zn ratio from 10:0, 9:1, and 8:2 to 7:3.

##### Material Characterization

The XRD patterns were recorded in a 2*θ* range of 5–60°. The morphology and composition were investigated by the Zeiss Sigma HD scanning electron microscopy and Thermofisher Themis Z HR‐TEM. The optical performances were investigated by UV3600 Shimadzu. The steady‐state PL spectra were recorded on a Fluorolog‐Tau3 fluorescence spectrophotometer with an excitation wavelength of 350 nm. IPCE was tested with the Newport series, as detailed in Supporting Information.

##### PEC Performance Characterization

The PEC performance was tested by a CHI660E electrochemical station. The applied electrolyte consisted of 1 m NaOH. Before the test, the system was bubbled with N_2_ to remove the dissolved gas. The photoanode potential was converted into the RHE potential, according to Nernst's equation.^[^ ^43^, ^44^ ^]^

(5) $$E_{\text{RHE}}=E_{\text{Ag/AgCl}}+0.059\text{pH}+E_{\text{Ag}/\text{AgCl}}^{0}$$

where *E*_RHE_ is the converted potential versus RHE, *E*_Ag/AgCl_ is the measured potential versus the Ag/AgCl electrode, and *E* ^°^_Ag/AgCl_ =0.1976 V at 25 °C.

## Conflict of interest

The authors declare no conflict of interest.

## Data Availability Statement

Research data are not shared.

## Supporting information

Supplementary Material
