# Supplementary material for: Multigraded Heterojunction Hole Extraction Layer of ZIF‐Co x Zn1−x on Co3O4/TiO2 Skeleton for a New Photoanode Architecture in Water Oxidation
Source: Small Sci. 2021 Feb 24;1(4):2000033. doi: 10.1002/smsc.202000033 (PMC11935825; doi:10.1002/smsc.202000033)
Supplement: Supplementary file 1 — Supplementary Material [file SMSC-1-2000033-s001.docx]

***Supporting Information***

**Multi-graded heterojunction hole extraction layer of ZIF-Co_x_Zn_1-x_ on Co_3_O_4_/TiO_2_ skeleton for a new photoanode architecture in water oxidation**

Rui Tang^1,2^, Lizhuo Wang^2^, Meihui Ying^2^, Wenjie Yang^2^, Amanj Kheradmand^3^, Yijiao Jiang^3^, Zhiyun Li^4^, Yi Cui^4^, Rongkun Zheng^1, *^, Jun Huang^2, *^

Dr. R. Tang, Prof. R. Zheng

School of Physics, Sydney Nano Institute, The University of Sydney, Sydney, NSW 2006, Australia

E-mail: rongkun.zheng@sydney.edu.au

Dr. R. Tang, Mr. L. Wang, Ms. M. Ying, Mr. W. Yang, Prof. J. Huang
School of Chemical and Biomolecular Engineering, Sydney Nano Institute, The University of Sydney, NSW 2037, Australia

E-mail: jun.huang@sydney.edu.au

Mr. A. Kheradmand, Prof. Y. Jiang
School of Engineering, Macquarie University, Sydney, NSW 2109, Australia

Ms. Zhiyun Li, Prof. Y. Cui

Vacuum Interconnected Nanotech Workstation, Suzhou Institute of Nano–Tech and Nano-Bionics, the Chinese Academy of Sciences, Suzhou 215123, China

1. **Supporting Text**

**Calculation of carrier density (N*d*) through the Mott-Schottky plots:**

The carrier density (N*d*) of the obtained photoanode could be determined through the following equation:

$Nd=\frac{2}{e_{0}\varepsilon\varepsilon_{0}}\left[\frac{d\left(\frac{1}{C^{2}}\right)}{dv}\right]^{-1}$ (Eq. S1),

Where *ε_0_* is the permittivity of free space (8.86×10^-12^ F/m); ε is the dielectric constant; *e_0_* is electron charge (1.6×10^-19^ C) ^[1-3]^.

**Calculation of applied bias photon-to-current conversion efficiency (ABPE):**

The ABPE of the obtained photoanodes could be calculated through the following equation ^[4]^:

$ABPE=\frac{J\times\left(1.23-V_{app}\right)}{J_{inco\min g}}\times100\%$ (Eq. S2),

where *J* is the photocurrent density arisen from the linear scan voltammetry plots; *V_app_* is the applied test-voltage; *J_incoming_* is the incoming light capacity.

**Calculation of the electrochemical active surface area (ECSA)**

To calculate the ECSA of the obtained photoanodes, the double-layer capacitance (*C**_dl_*) is tested with cyclic voltammetry (CV) in a non-Faradaic region of 0.7-0.9 V *vs.* RHE at scan rates of 20, 50, 100, 150, 200, 250, and 300 mV s^-1^. The current difference (*ΔI*) of the anodic charging current (*I_a_*) and cathodic charging current (*I_c_*) is plotted against the scan rate, and the linear slope is twice of *C_dl_*. The ECSA is calculated from *C_dl_* according to the equation ^[5-6]^:

ECSA = *C_dl_* / *C_s_*  (Eq. S3),

where *C_s_* is the specific capacitance of the sample.

**Calculation of incident photon-to-current conversion efficiency (IPCE):**

The IPCE of the obtained photoanodes could be calculated through the following equation ^[4]^:

**$IPCE=\frac{\left[\left(\frac{1240}{\lambda}\right)\times\left(J_{light}-J_{dark}\right)\right]}{J_{inco\min g}}\times100\%$**  (Eq. S4),

Where *λ* is the test-wavelength and *J_light_* and *J_dark_* are the photocurrent density under illumination and dark condition. The IPCE is measured under monochromatic irradiations from a 150 W Xe lamp with monochromator at 1.23 V *vs.* RHE.

**Calculation of the decay lifetime of photovoltage-time (*V*-t) plots**

The corresponding decay lifetime is calculated according to the bi-exponential function with two time constants ^[7]^:

*y* (*t*) = *A*_0_ + *A*_1_e^-^*^t/τ^*^1^ + *A*_2_*e^-t/τ^*^2^ (Eq. S5),

*τ*_m_ = (*τ*_1_*τ*_2_) / (*τ*_1_ + *τ*_2_) (Eq. S6),

Where *τ*_m_ is the harmonic mean of the lifetime and the total half-life is log (2 × *τ*_m_).

**Calculation of the charge injection and charge separation efficiency**

Photocurrent density arisen from PEC water oxidation could be described by the following equation ^[8-9]^:

*J _H2O_* = *J _absorbed_* × *η* _charge separation_ × *η* _charge injection_  (Eq. S7),

Where *J _absorbed_* is the photocurrent density integrated from the complete absorbed incident light, which is a constant with the illumination source; *η*_charge_ _separation_ is the charge separation efficiency of the photogenerated charge carriers; *η*_charge injection_ is the charge injection efficiency from electrode to electrolyte. To obtain detailed information for the efficiencies in each process, the hole scavenger (Na_2_SO_3_) is added into the electrolyte to reduce the energy loss in the surface water oxidation process (charge injection) due to its fast hole capture kinetics. The charge separation and charge injection efficiency can be calculated as follows:

*_Na2SO3_* = *J _absorbed_* × *η* _charge separation_  (Eq. S8),

*η* _charge separation_ = *J _Na2SO3_*/*J_absorbed_* (Eq. S9),

*η* _charge injection_ = *J _H2O_*/*J _Na2SO3_* (Eq. S10),

1. **Supporting Figures**


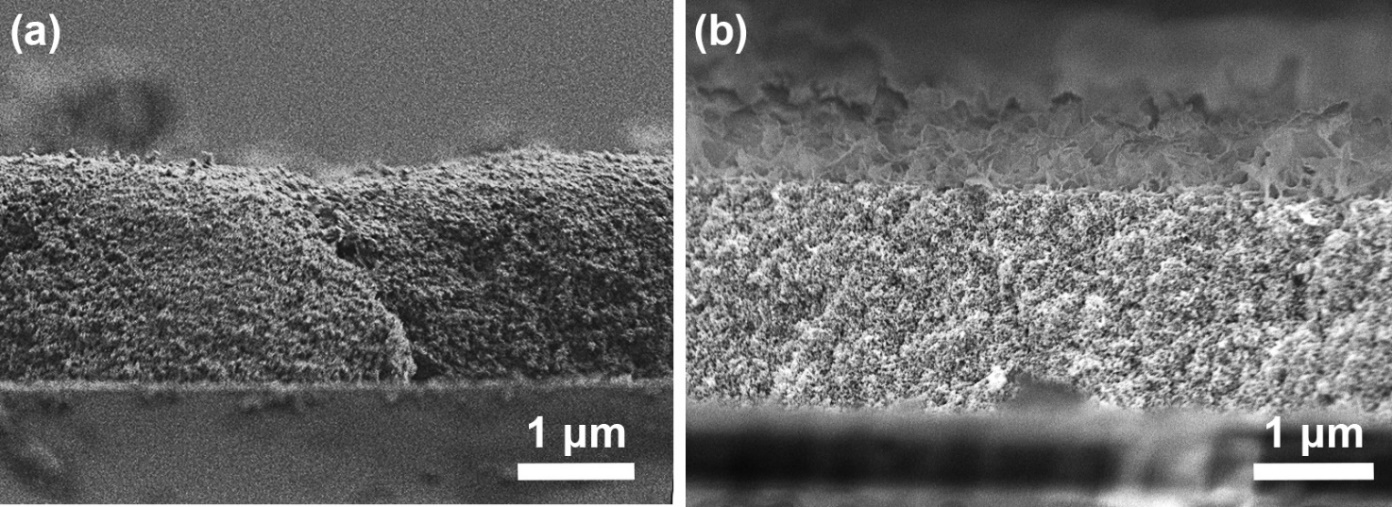


Figure S1. Cross-view SEM images of (a) TiO_2_ absorption layer and (b) network-like Co_3_O_4_/TiO_2_ photoanodes.


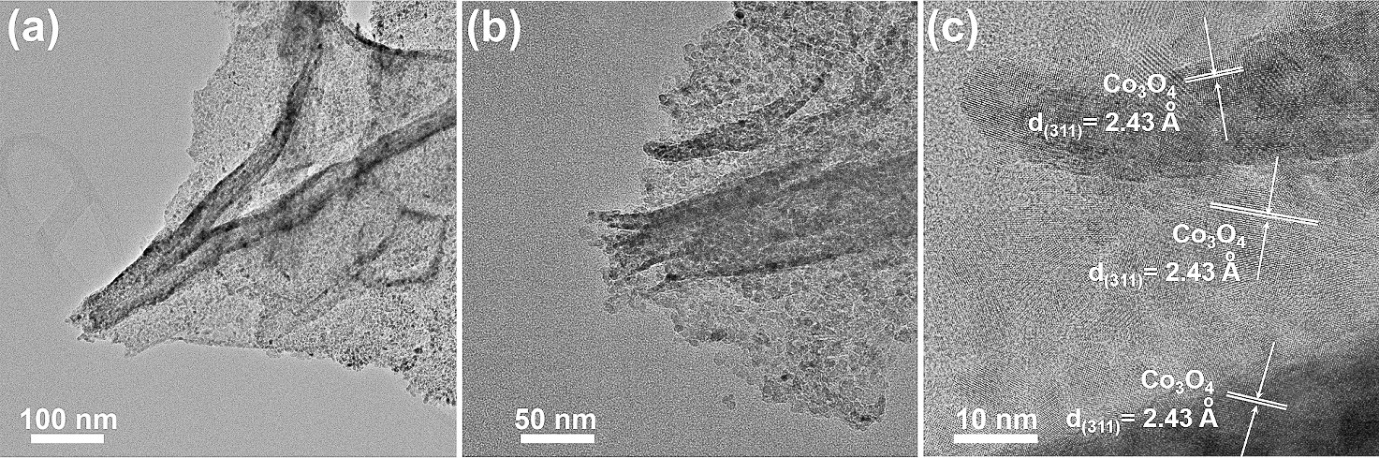


Figure S2.  (a-b) TEM images of Co_3_O_4_ skeleton. (c) HR-TEM image of Co_3_O_4_ skeleton. The d-spacing of 2.43 Å in HRTEM lattice image can be indexed to (311) plane of Co_3_O_4_.

From Figure S2a-b, it can be seen that the Co_3_O_4_ skeleton is composed of porous nanoparticle structure. This unique structure can be beneficial for the electrolyte infiltration. From the HR-TEM image, the marked d-spacing of 2.43 Å can be indexed to the (311) planes of Co_3_O_4_.


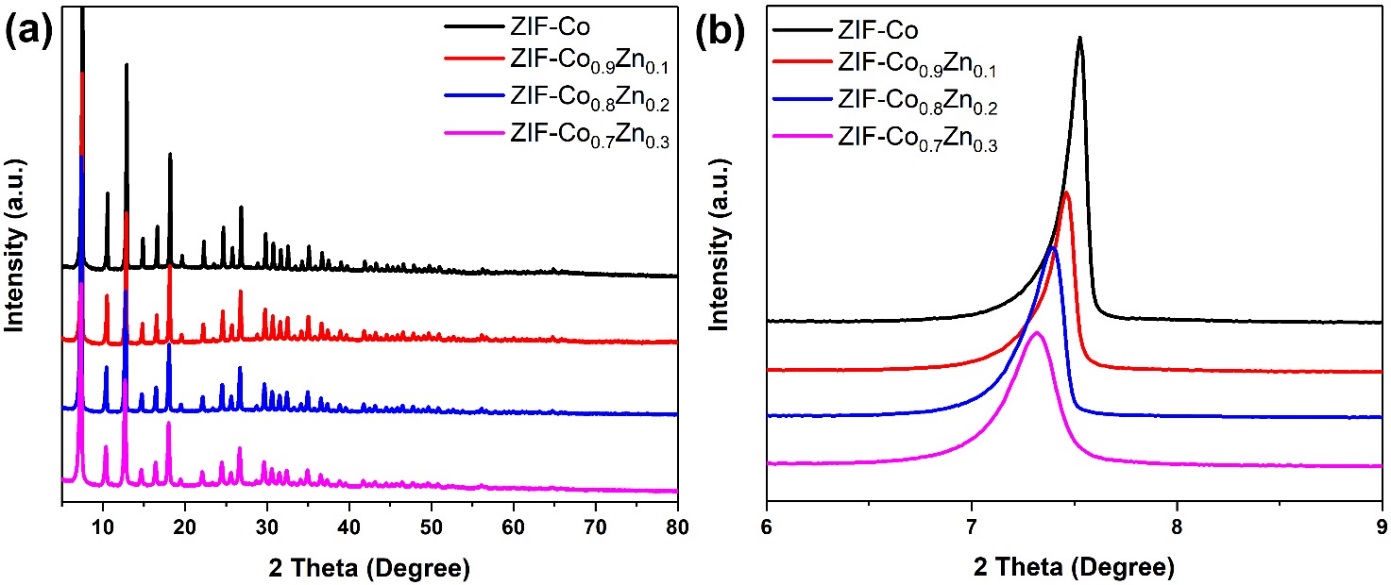


Figure S3. (a) XRD of ZIF-Co, ZIF-Co_0.9_Zn_0.1_, ZIF-Co_0.8_Zn_0.2_, and ZIF-Co_0.7_Zn_0.3_. (b) Magnified XRD patterns in the 2 Theta range of 6-9 degree.


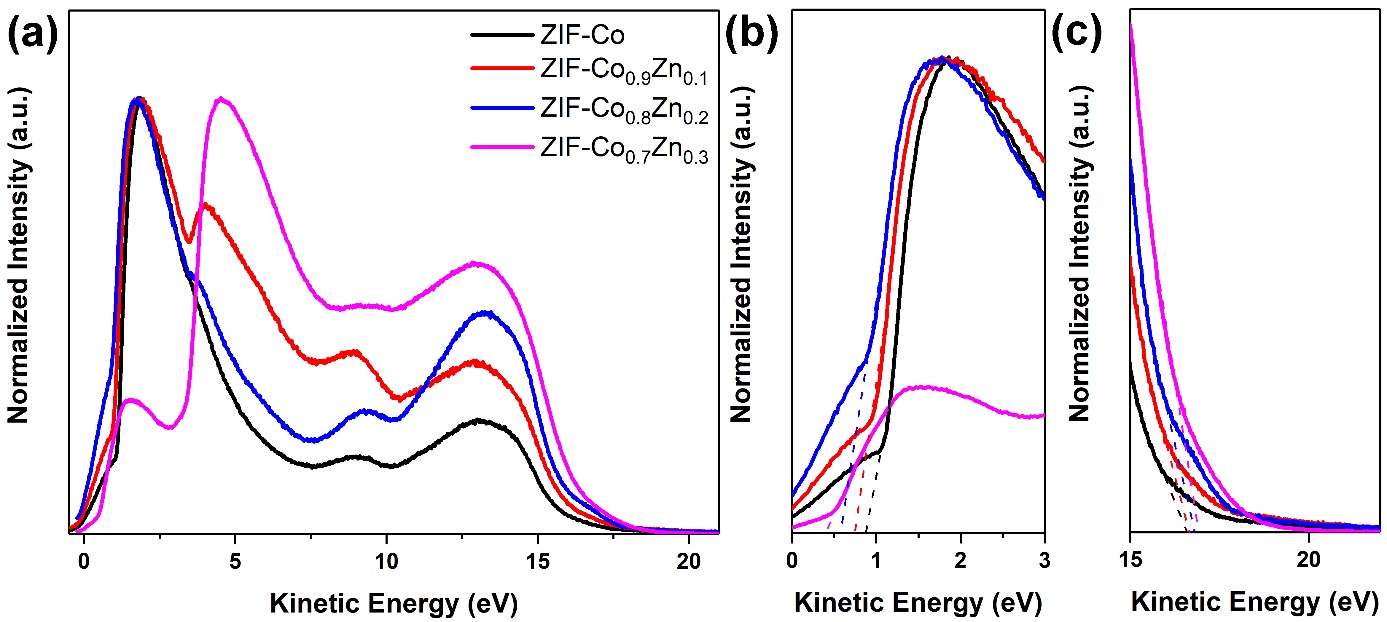


Figure S4. (a) Ultraviolet photoemission spectroscopy (UPS) of ZIF-Co*_x_*Zn*_1-x_* (*x*=1.0, 0.9, 0.8, 0.7) samples. (b) and (c) are partial enlarged views of Figure (a).


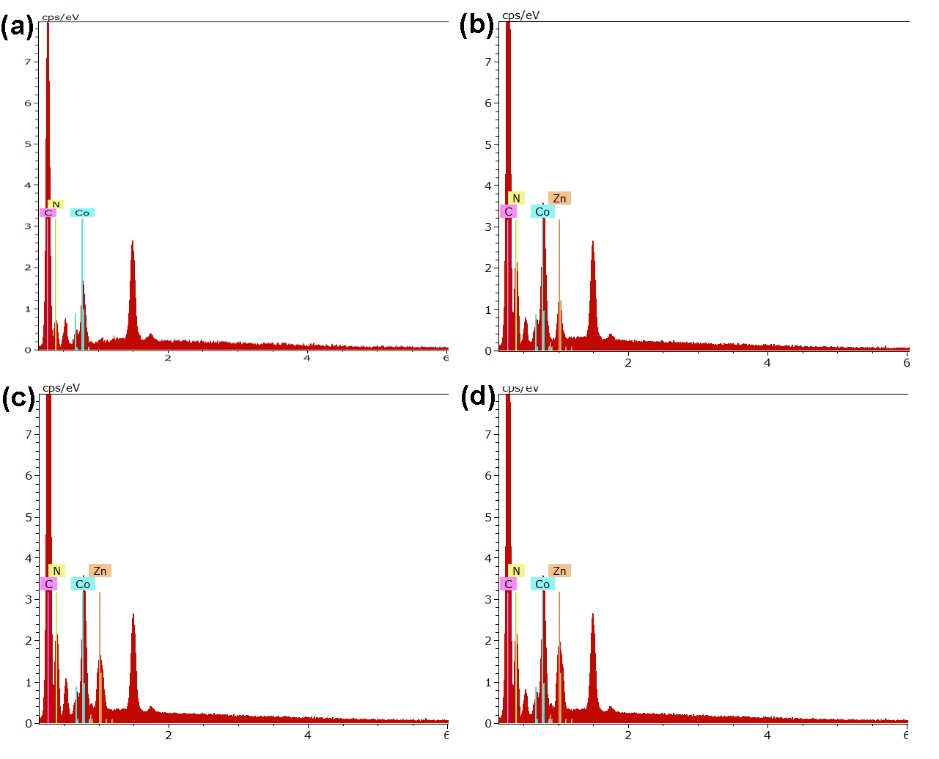


Figure S5. EDS spectra of the ZIF-Co*_x_*Zn*_1-x_* (*x*=1.0, 0.9, 0.8, 0.7) samples.

Through the EDS spectra, the relative Zn/Co ratios of obtained ZIF-Co*_x_*Zn*_1-x_* (*x*=0.9, 0.8, 0.7) samples are calculated to be 11%, 25%, and 41%. Through inductive coupled plasma (ICP) emission spectrometer, the Zn/Co ratios of obtained ZIF-Co*_x_*Zn*_1-x_* (*x*=0.9, 0.8, 0.7) samples are further calculated to be 10.7 %, 27.5 %, and 35.6 %, respectively. Therefore, considering the EDS and ICP results, it can be concluded that the Co/Zn ratio in the obtained ZIF-Co*_x_*Zn*_1-x_* (*x*=1.0, 0.9, 0.8, 0.7) samples can indeed be efficiently tailored by adjusting the precursor.


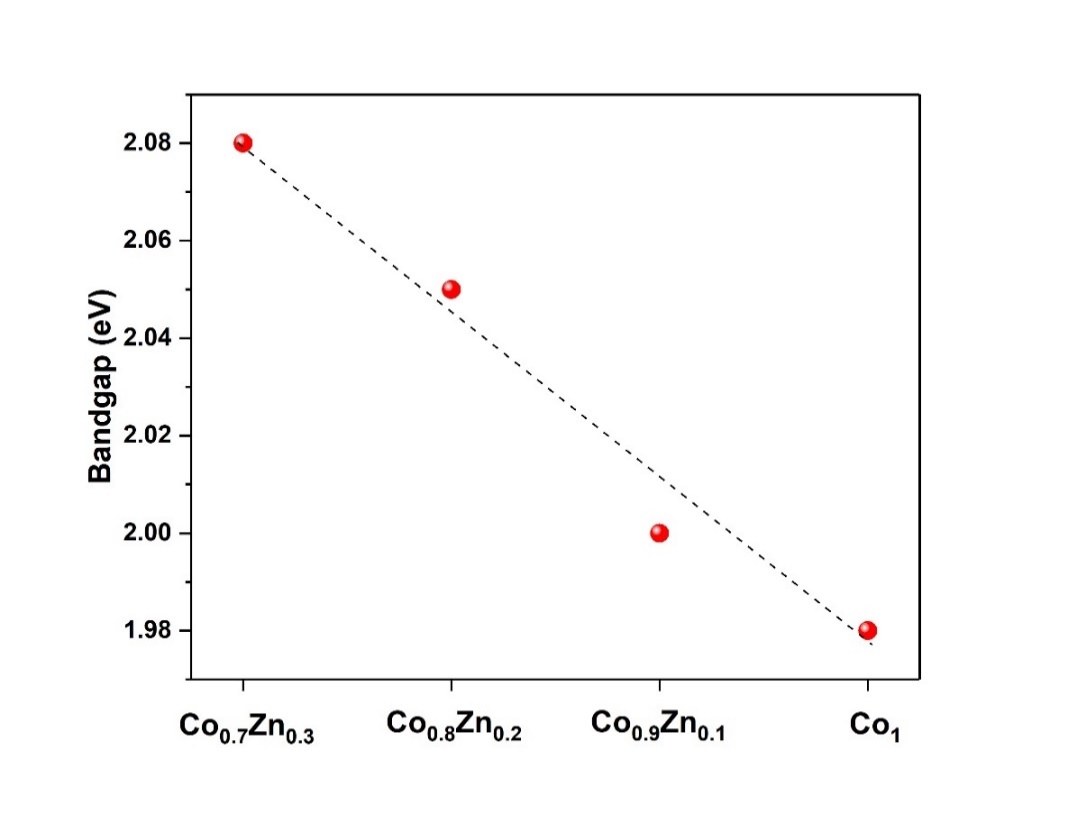


Figure S6. Co/Zn Ratio-bandgap curves of the as-prepared ZIF-Co_x_Zn_1-x_.


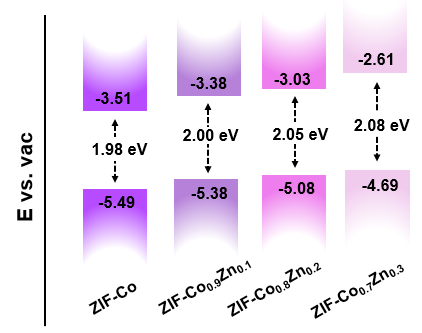


Figure S7. Relative energy alignment structure of ZIF-Co, ZIF-Co_0.9_Zn_0.1_, ZIF-Co_0.8_Zn_0.2_, and ZIF-Co_0.7_Zn_0.3_.


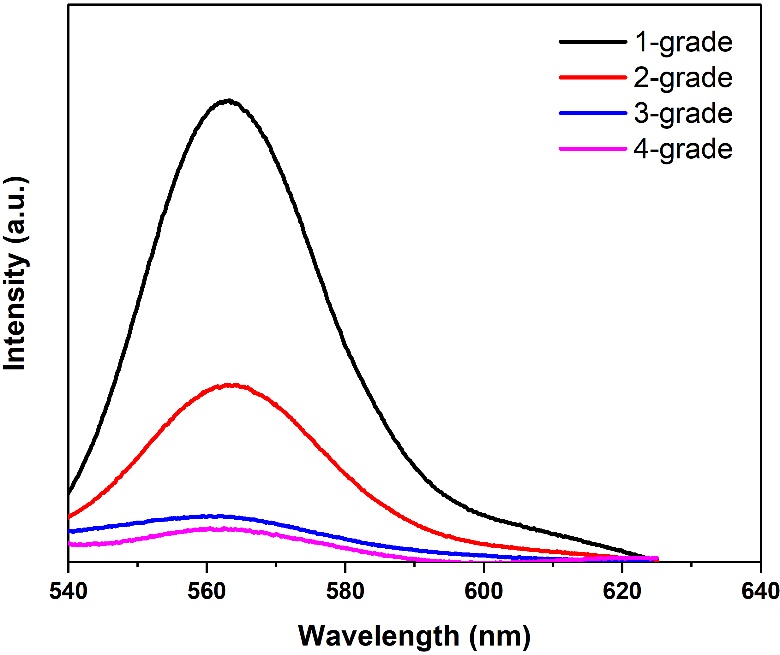


Figure S8. Photoluminescence spectra of 1-grade, 2-grade, 3-grade, and 4-grade samples.


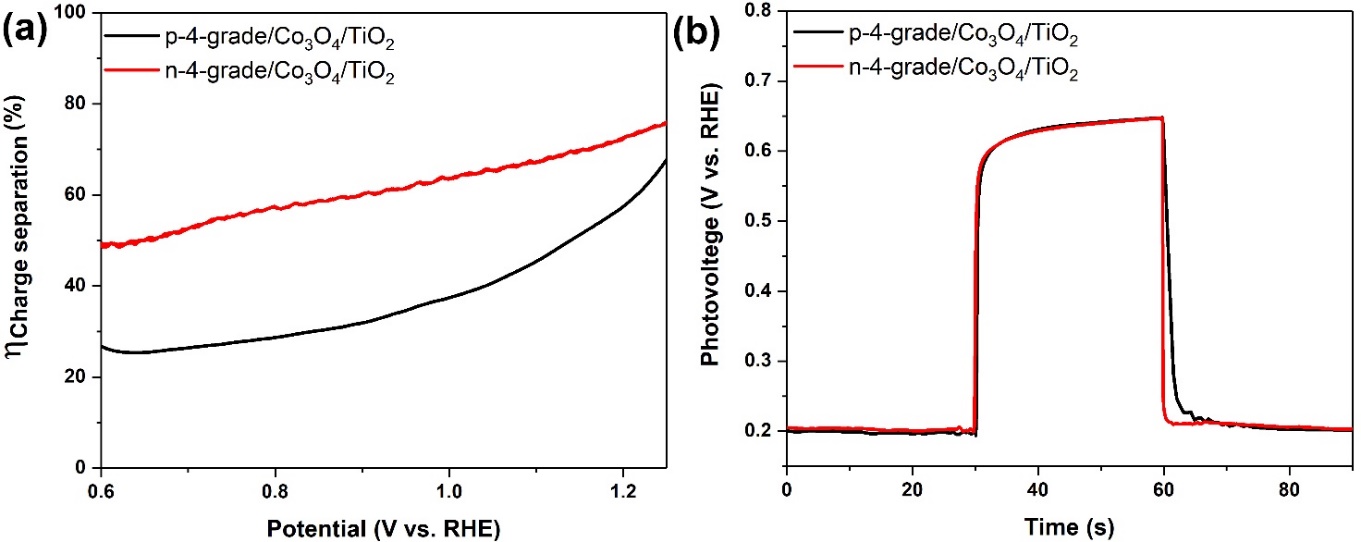


Figure S9 (a) Charge separation efficiency plots and (b) photovoltage-decay plots of p-4-grade/Co_3_O_4_/TiO_2_ and n-4-grade/Co_3_O_4_/TiO_2_.


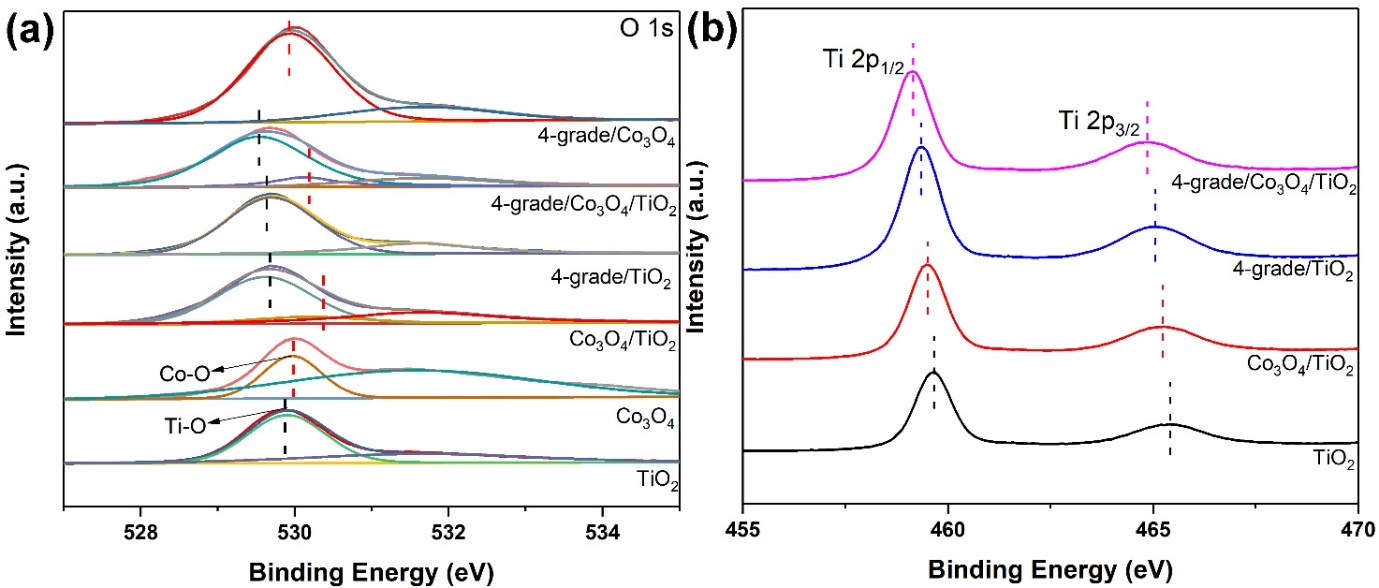


Figure S10. X‐ray photoelectron spectroscopy spectra of (a) O 1s of TiO_2_, Co_3_O_4_, Co_3_O_4_/TiO_2_, 4-grade/TiO_2_, 4-grade/Co_3_O_4_/TiO_2_, and 4-grade/Co_3_O_4_ sample, (b) Ti 2p of pristine TiO_2_, Co_3_O_4_/TiO_2_, 4-grade/TiO_2_, and 4-grade/Co_3_O_4_/TiO_2_


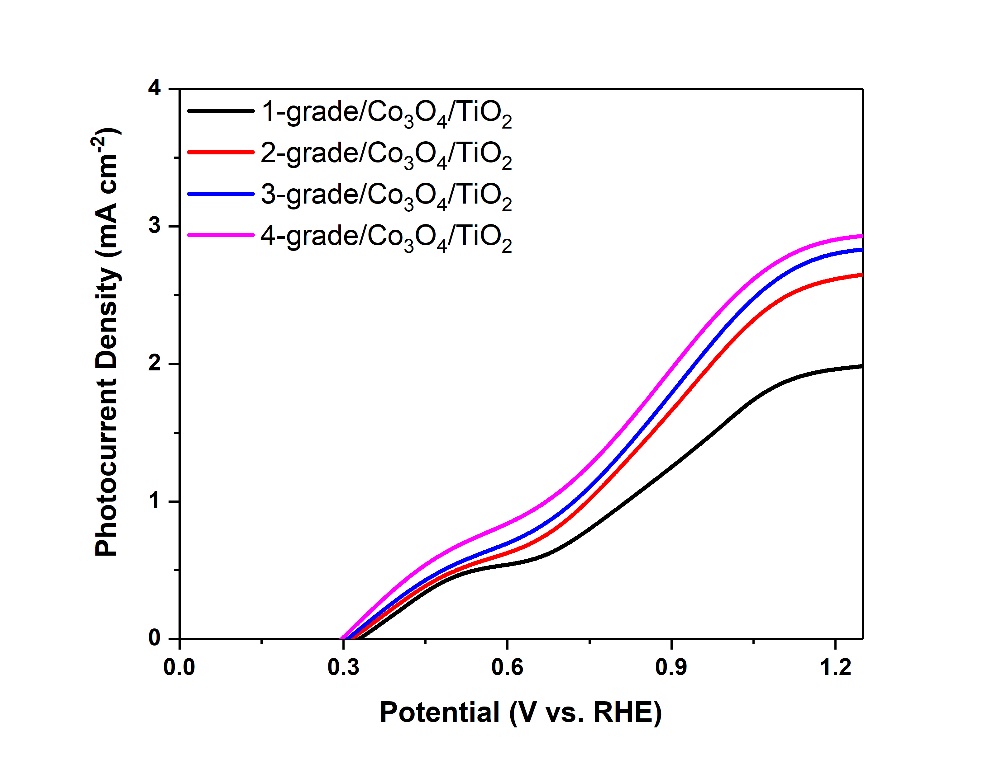


Figure S11. Linear sweep voltammogram curves of multi-GHJ samples.

The LSV curves of photoanode with different grade were also tested. It can be evidenced that, with the grade increase, the photocurrent shows an increasing trend. At last the 4-grade sample shows the largest photocurrent density.


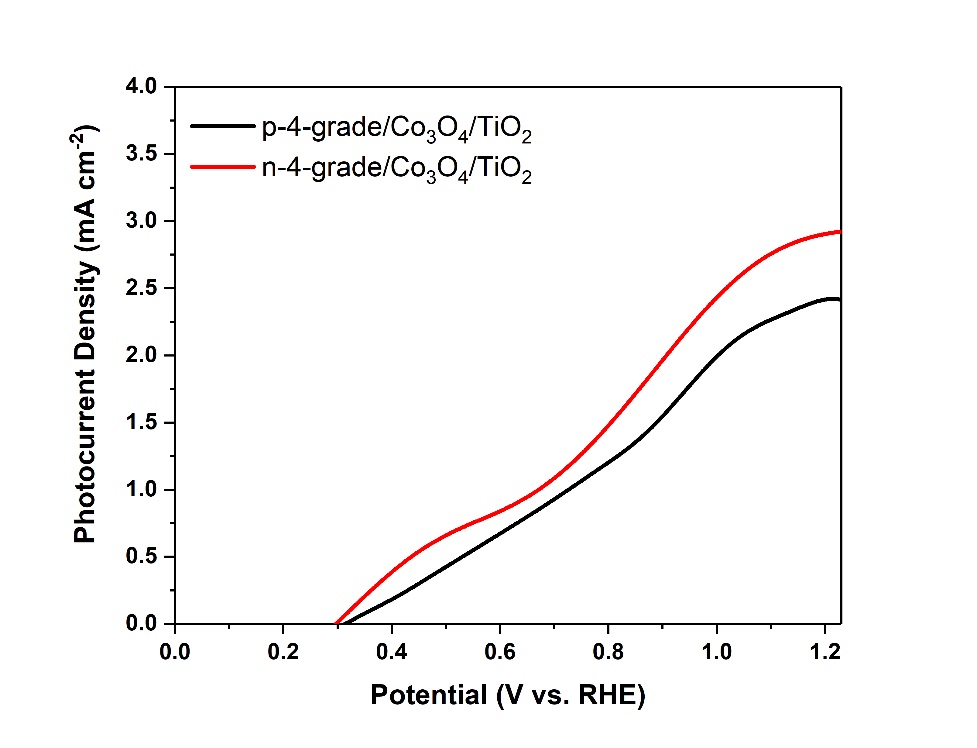


Figure S12. Linear sweep voltammogram curves of p-4-grade/Co_3_O_4_/TiO_2_ and n-4-grade/Co_3_O_4_/TiO_2_.


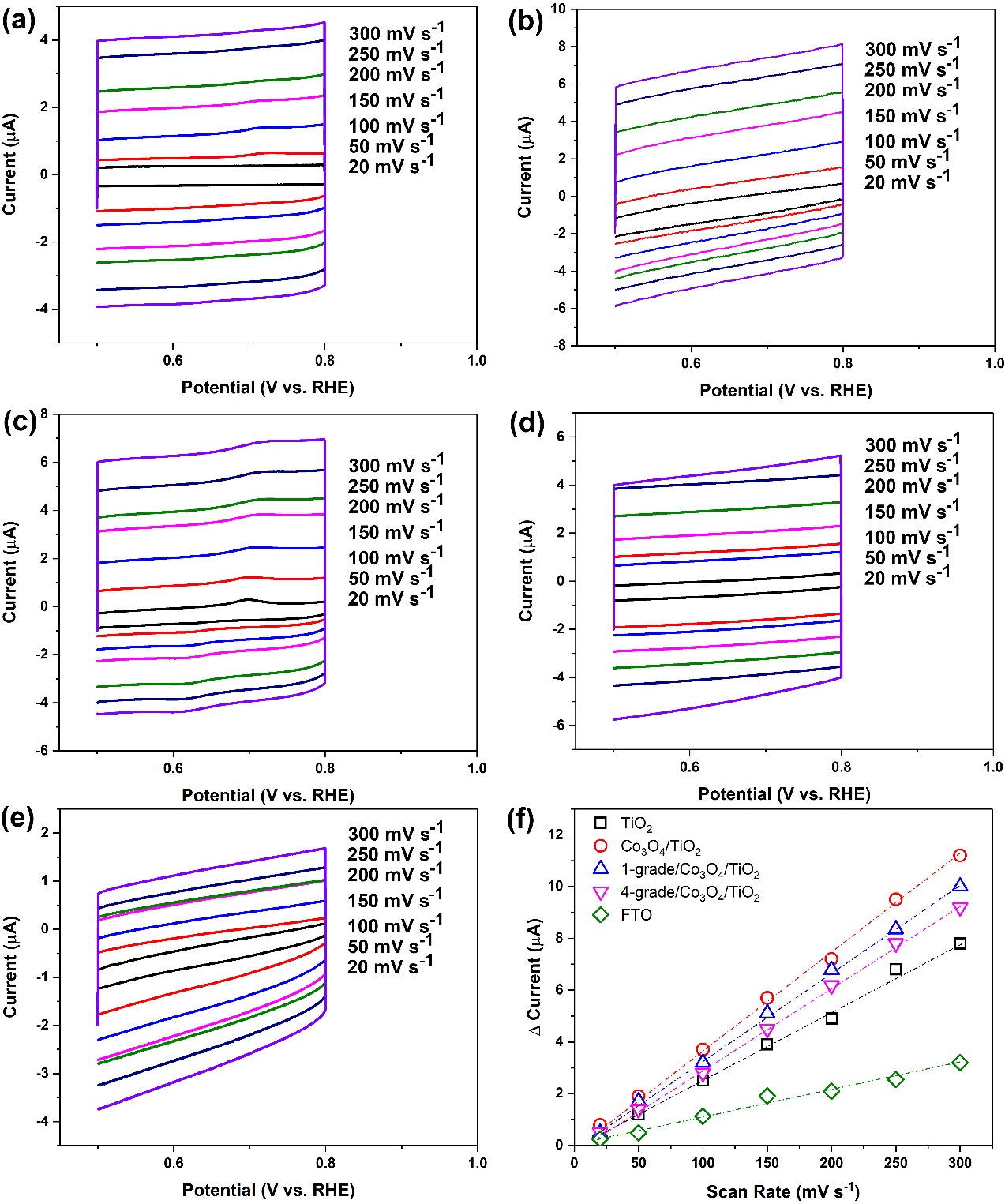


Figure S13. CV curves measured in a non-Faradaic region of 0.5-0.8 V at various scan rates for (a) TiO_2_, (b) Co_3_O_4_/TiO_2_, (c) 1-grade/Co_3_O_4_/TiO_2_, (d) 4-grade/Co_3_O_4_/TiO_2_, and (e) FTO with a geometric area of 4 cm^2^ , respectively. (f) Charging current differences (ΔI = Ia - Ic) against scan rate for TiO_2_, Co_3_O_4_/TiO_2_, 1-grade/Co_3_O_4_/TiO_2_, 4-grade/Co_3_O_4_/TiO_2_ and FTO respectively.

The ECSA of the obtained TiO_2_, Co_3_O_4_/TiO_2_, 1-grade/Co_3_O_4_/TiO_2_, and 4-grade/Co_3_O_4_/TiO_2_ photoanodes are calculated according to Eq. S3. According to the testing result, the ECSA of the TiO_2_, Co_3_O_4_/TiO_2_, 1-grade/Co_3_O_4_/TiO_2_, and 4-grade/Co_3_O_4_/TiO_2_ photoanodes are calculated to be 2.43, 3.50, 3.13 and 2.87 cm^2^, respectively.


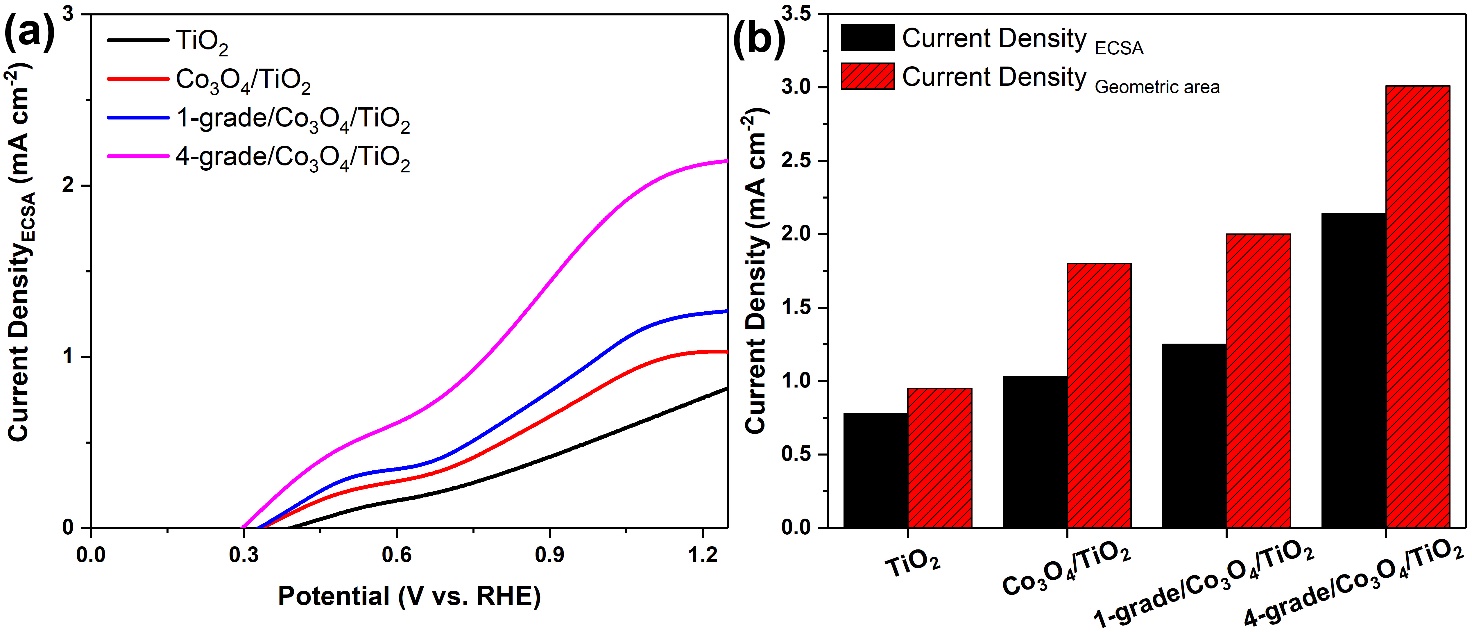


Figure S14. (a) ECSA normalized photocurrent density, (b) comparisons of the photocurrent density normalized by the geometrical area and the ECSA at 1.23 V for TiO_2_, Co_3_O_4_/TiO_2_, 1-grade/Co_3_O_4_/TiO_2_, and 4-grade/Co_3_O_4_/TiO_2_, respectively.


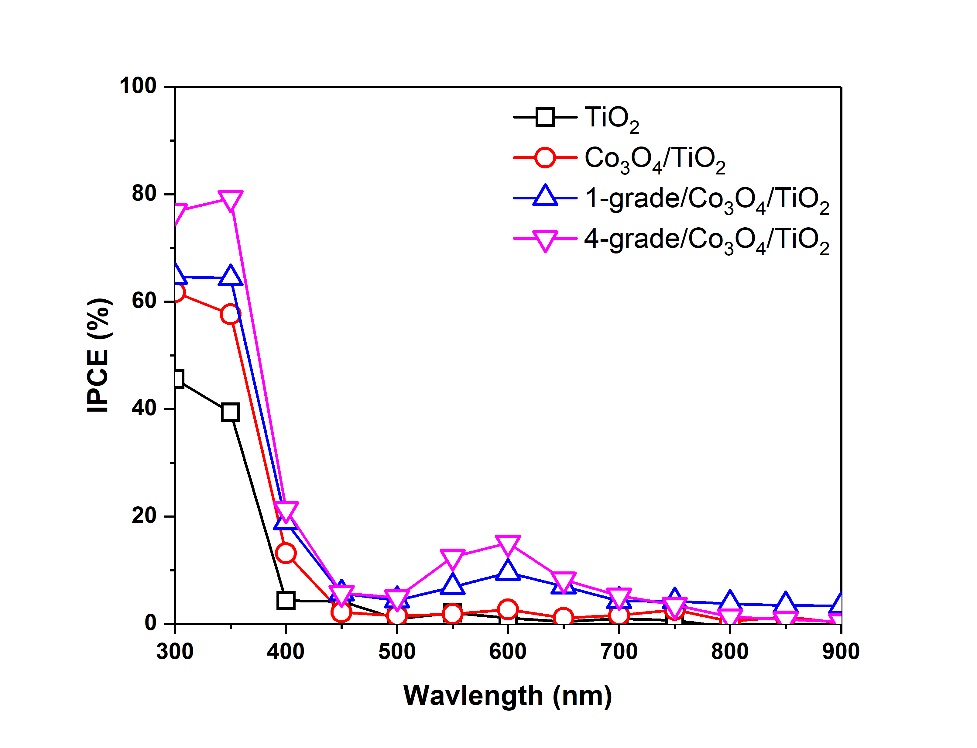


Figure S15. Incident photon‐to‐current conversion efficiency (IPCE) of obtained TiO_2_, Co_3_O_4_/TiO_2_, 1-grade/Co_3_O_4_/TiO_2_ and 4-grade/Co_3_O_4_/TiO_2_ photoanodes.


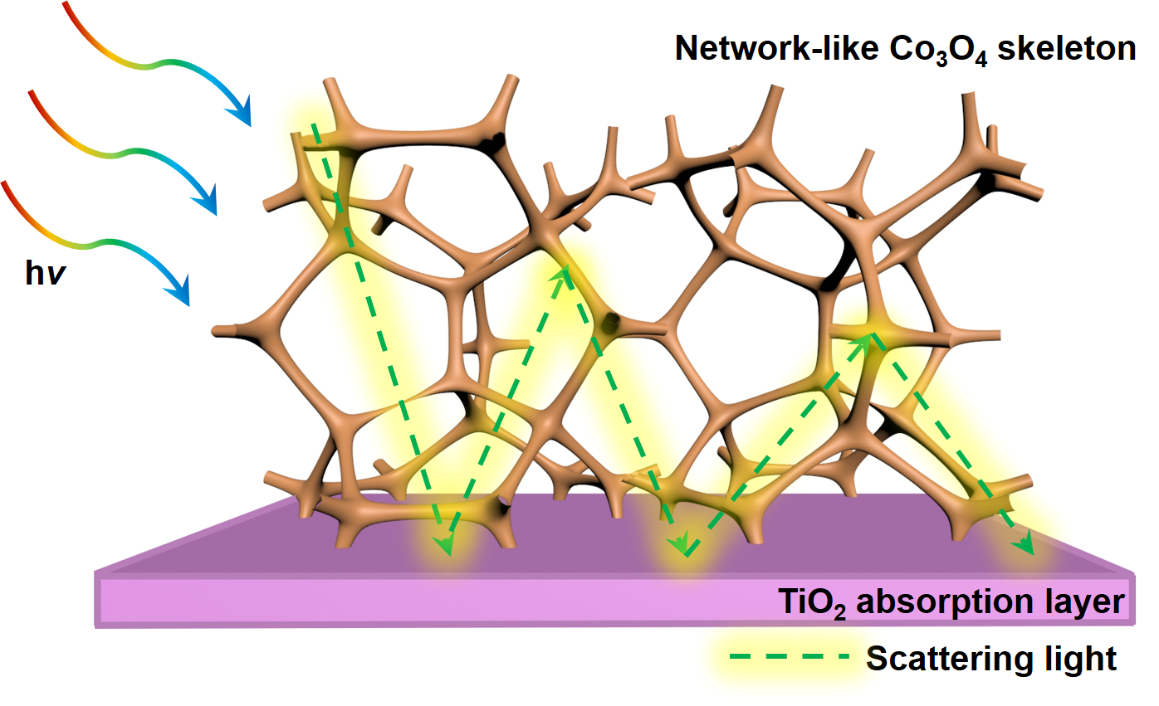


Figure S16. Schematic illustration of incident light traveling path within the network-like Co_3_O_4_/TiO_2_ photoanode.


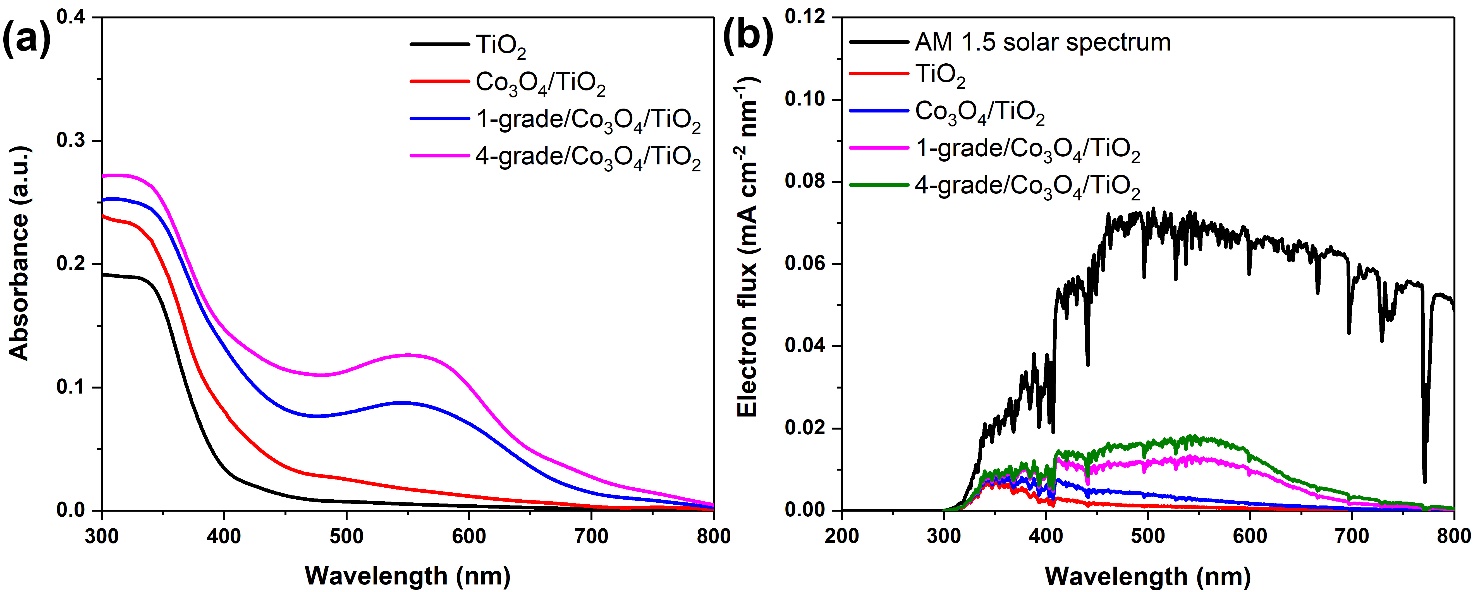


Figure S17. (a) UV-vis absorbance spectra and (b) electron flux of TiO_2_, Co_3_O_4_/TiO_2_, 1-grade/Co_3_O_4_/TiO_2_ and 4-grade/Co_3_O_4_/TiO_2_ photoanodes.

From the UV-vis absorption, it can be evidenced that after the growth of network-like Co_3_O_4_ skeleton, besides slightly broadened optical response range, the light harvesting capability is also greatly enhanced. This should be attributed to the multiple light scattering processes within the network-like Co_3_O_4_ skeleton. Due to the multi-light scattering effect, the incident light traveling length is obviously enhanced, which leads to an enhanced light capture capability.

After further decorating ZIF-Co*_x_*Zn_1_*_-x_*, an extra absorption peak shows in the wavelength range of 500-650 nm, which should be attributed to the optical response of ZIF-Co*_x_*Zn_1-_*_x_*. From the electron flux image, the optical response of ZIF-Co*_x_*Zn_1-_*_x_* is more clear.


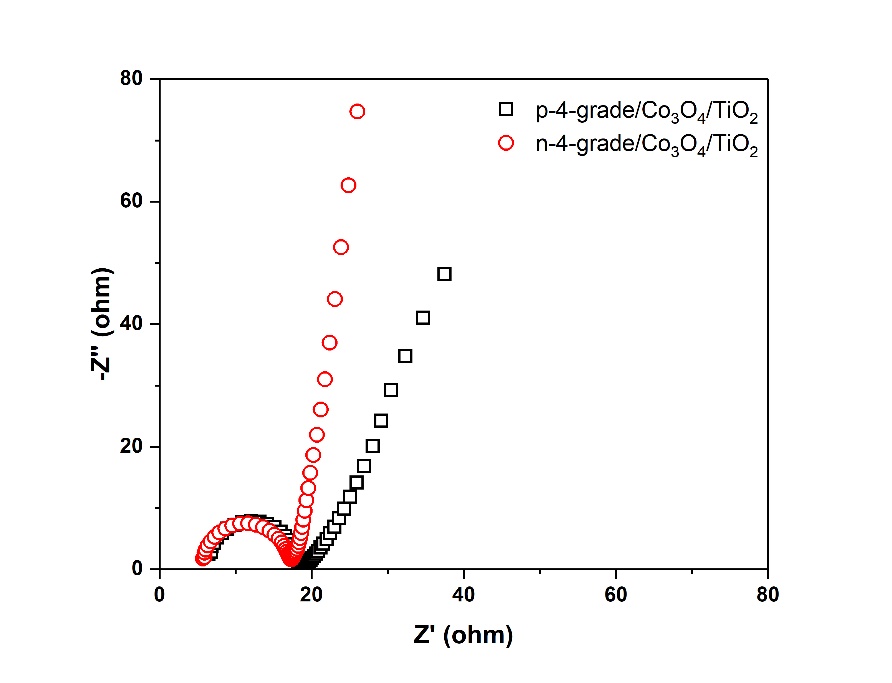


Figure S18. Electrochemical impedance spectra of p-4-grade/Co_3_O_4_/TiO_2_ and n-4-grade/Co_3_O_4_/TiO_2_.


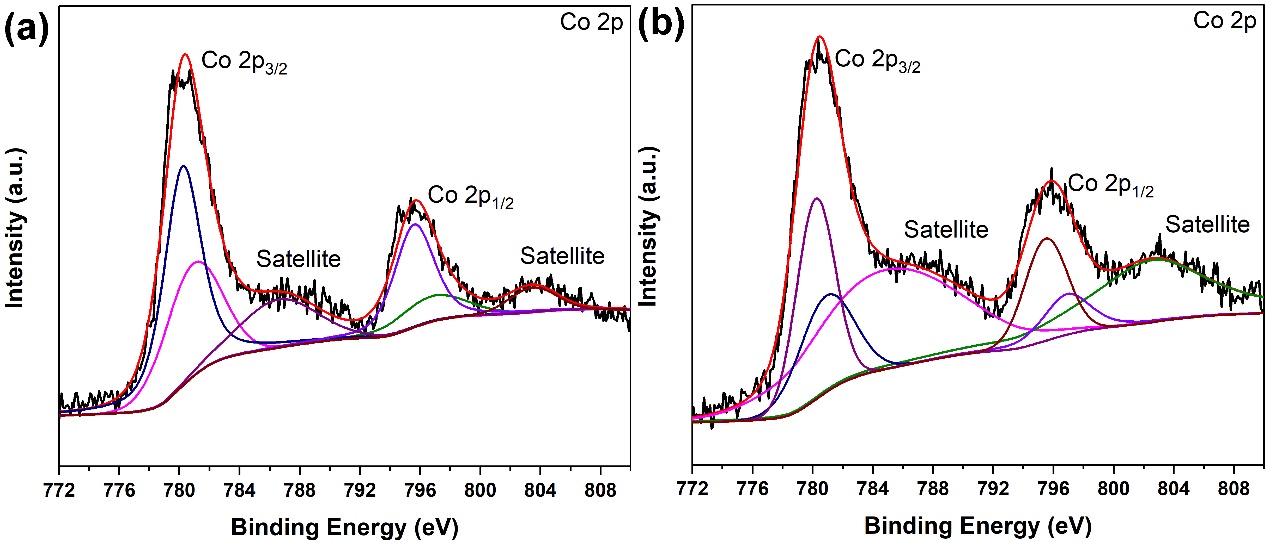


Figure S19. The XPS Co 2p spectra of fresh and used photoanodes.


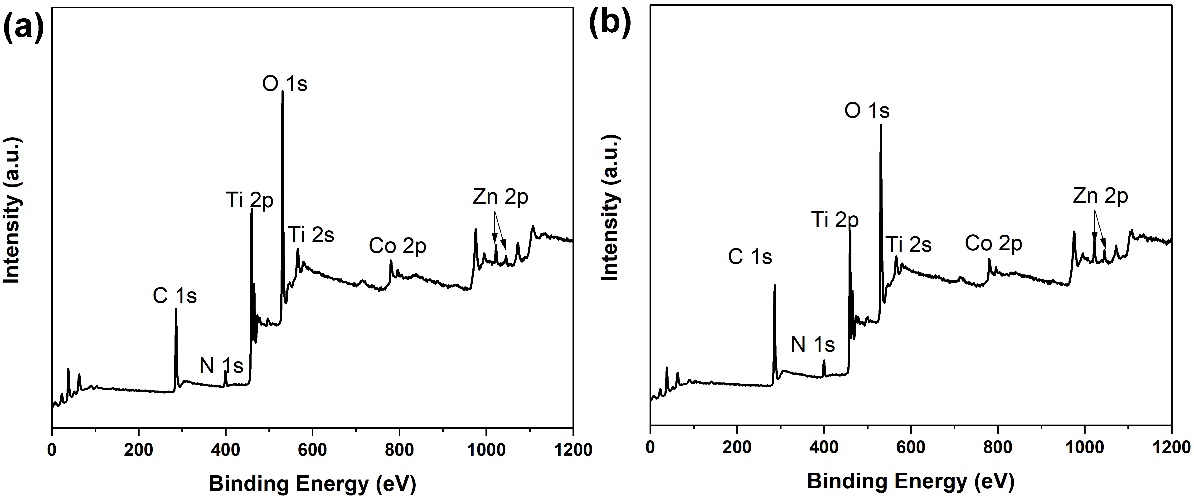


Figure S20. The survey XPS spectra of fresh and used photoanodes.


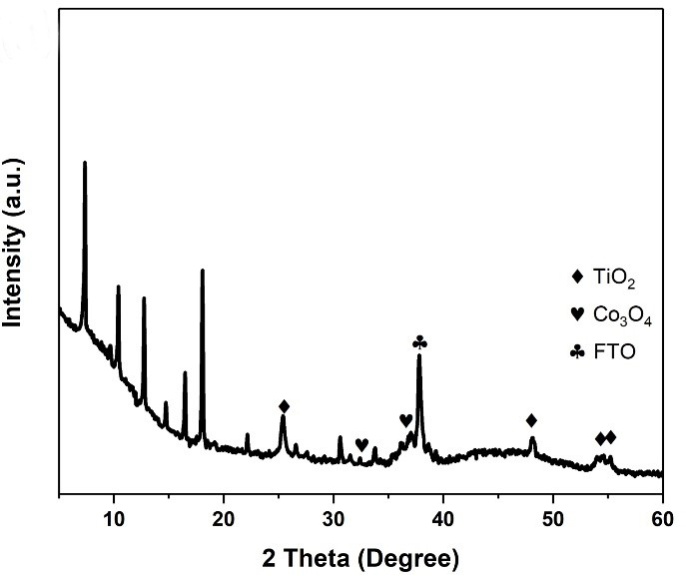


Figure S21. XRD pattern of 4-grade/Co_3_O_4_/TiO_2_ after 1-h PEC reaction.


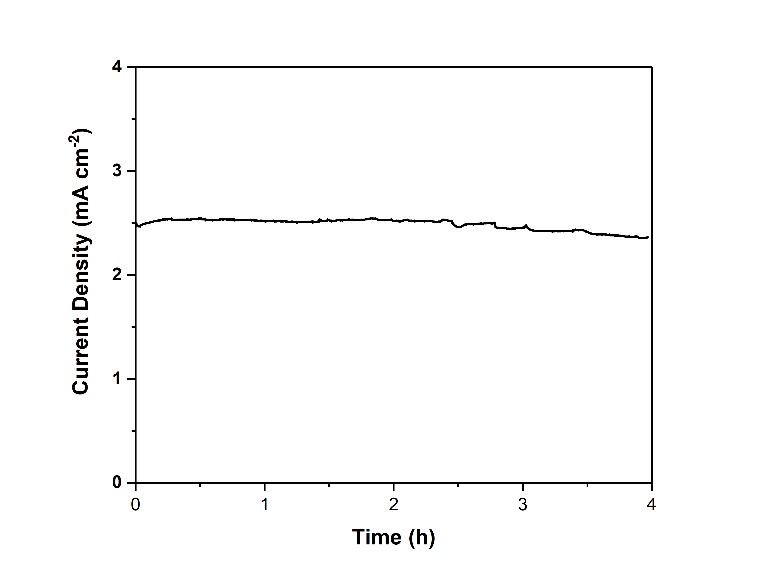


Figure S22. Chronoamperometric I-t curves of 4-grade/Co_3_O_4_/TiO_2_ at a bias potential of 1.0 V versus RHE.

The 4-grade/Co_3_O_4_/TiO_2_ sample is further tested at a bias potential of 1.0 V versus RHE for 4 hours to investigate the stability of the photoanode material. During the 1-h I-t testing, the photocurrent density of 4-grade/Co_3_O_4_/TiO_2_ sample shows slight decreasing of about 5.3 %, which further confirms the stability of the photoanode material.

1. **Supporting Table**

Table S1. A comparison study of the 4-grade/Co_3_O_4_/TiO_2_ in this work and some latest high performance photoanodes towards PEC water oxidation

| Sample | Photocurrent density | η_charge separation_ ×  η_charge injection_ | Reference |
| --- | --- | --- | --- |
| BiVO_4_-N/C-CoPOM | 3.30 mA cm^-2^  (1.23 V vs. RHE) | 58.9 % | ^[10]^ |
| Lu_2_O_3_/BiVO_4_ | 1.72 mA cm^-2^  (1.23 V vs. RHE) | 36.2 % | ^[11]^ |
| Surface disorder treated TiO_2_ nanorod | 1.18 mA cm^-2^  (1.23 V vs. RHE) | 52.3 % | ^[12]^ |
| Phosphorus-doped TiO_2_ | 2.5 mA cm^-2^  (1.23 V vs. RHE) | 56.1% | ^[13]^ |
| TiO_2_-SrTiO_3_ core-shell nanowire | 1.43 mA cm^-2^  (1.23 V vs. RHE) | 78.9 % | ^[14]^ |
| TiO_2_-BaTiO_3_ core-shell nanowire | 1.30 mA cm^-2^  (1.23 V vs. RHE) | 79.8 % | ^[15]^ |
| Z-scheme ZnO/TiO_2_ | 2.75 mA cm^-2^  (1.23 V vs. RHE) | 78.3 % | ^[16]^ |
| CQDs-H/TiO_2_ | 3.0 mA cm^-2^  (1.23 V vs. RHE) | - | ^[17]^ |
| SN1-TiO_2_ NRs | 2.82 mA cm^-2^  (1.23 V vs. RHE) | - | ^[18]^ |
| Ta_3_N_5_-15-15 | 3.55 mA cm^-2^  (1.23 V vs. RHE) | 41.2 % | ^[19]^ |
| FeOOH/rGO/BiVO_4_ | 3.25 mA cm^-2^  (1.23 V vs. RHE) | 67.1 % | ^[20]^ |
| 4-grade/Co_3_O_4_/TiO_2_ | 2.91 mA cm^-2^  (1.23 V vs. RHE) | 73.3 % | This work |

1. **References**

[1] J. R. Swierk, K. P. Regan, J. Jiang, G. W. Brudvig, C. A. Schmuttenmaer, *ACS Energy Lett.* **2016**, 1, 603.

[2] H.-i. Kim, D. Monllor-Satoca, W. Kim, W. Choi, *Energy Environ. Sci.* **2015**, 8, 247.

[3] W. Q. Fang, Z. Huo, P. Liu, X. L. Wang, M. Zhang, Y. Jia, H. Zhang, H. Zhao, H. G. Yang, X. Yao, *Chem. - Eur. J.* **2014**, 20, 11439.

[4] S. Shen, S. A. Lindley, X. Chen, J. Z. Zhang, *Energy Environ. Sci.* **2016**, 9, 2744.

[5] C. C. McCrory, S. Jung, J. C. Peters, T. F. Jaramillo, *J. Am. Chem. Soc.* **2013**, 135, 16977.

[6] F. Ning, M. Shao, S. Xu, Y. Fu, R. Zhang, M. Wei, D. G. Evans, X. Duan, *Energy Environ. Sci.* **2016**, 9, 2633.

[7] Y.-C. Pu, G. Wang, K.-D. Chang, Y. Ling, Y.-K. Lin, B. C. Fitzmorris, C.-M. Liu, X. Lu, Y. Tong, J. Z. Zhang, Y.-J. Hsu, Y. Li, *Nano Lett.* **2013**, 13, 3817.

[8] J. Zhang, X. Jin, P. I. Morales-Guzman, X. Yu, H. Liu, H. Zhang, L. Razzari, J. P. Claverie, *ACS nano* **2016**, 10, 4496.

[9] Y. Qiu, W. Liu, W. Chen, G. Zhou, P.-C. Hsu, R. Zhang, Z. Liang, S. Fan, Y. Zhang, Y. Cui, *Sci. Adv.* **2016**, 2, e1501764.

[10] K. Fan, H. Chen, B. He, J. Yu, *Chem. Eng. J.* **2019**, 123744.

[11] W. Zhang, D. Yan, X. Tong, M. Liu, *Adv. Funct. Mater.* **2018**, 28, 1705512.

[12] P. Yan, G. Liu, C. Ding, H. Han, J. Shi, Y. Gan, C. Li, *ACS Appl. Mater. Interfaces* **2015**, 7, 3791.

[13] D.-D. Qin, Q.-H. Wang, J. Chen, C.-H. He, Y. Li, C.-H. Wang, J.-J. Quan, C.-L. Tao, X.-Q. Lu, *Sustainable Energy Fuels* **2017**, 1, 248.

[14] F. Wu, Y. Yu, H. Yang, L. N. German, Z. Li, J. Chen, W. Yang, L. Huang, W. Shi, L. Wang, *Adv. Mater.* **2017**, 29, 1701432.

[15] W. Yang, Y. Yu, M. B. Starr, X. Yin, Z. Li, A. Kvit, S. Wang, P. Zhao, X. Wang, *Nano Lett.* **2015**, 15, 7574.

[16] T. Zhou, J. Wang, S. Chen, J. Bai, J. Li, Y. Zhang, L. Li, L. Xia, M. Rahim, Q. Xu, *Appl. Catal., B* **2020**, 267, 118599.

[17] Z. Liang, H. Hou, Z. Fang, F. Gao, L. Wang, D. Chen, W. Yang, *ACS Appl. Mater. Interfaces* **2019**, 11, 19167.

[18] D. M. Andoshe, K. Yim, W. Sohn, C. Kim, T. L. Kim, K. C. Kwon, K. Hong, S. Choi, C. W. Moon, S.-P. Hong, *Appl. Catal., B* **2018**, 234, 213.

[19] C. Shao, R. Chen, Y. Zhao, Z. Li, X. Zong, C. Li, *J. Mater. Chem. A* **2020**.

[20] G. Zeng, L. Hou, J. Zhang, J. Zhu, X. Yu, X. Fu, Y. Zhu, Y. Zhang, *ChemCatChem* **2020**, 12, 3769.
